# Circular RNA AFF4 modulates osteogenic differentiation in BM-MSCs by activating SMAD1/5 pathway through miR-135a-5p/FNDC5/Irisin axis

**DOI:** 10.1038/s41419-021-03877-4

**Published:** 2021-06-18

**Authors:** Chao Liu, An-Song Liu, Da Zhong, Cheng-Gong Wang, Mi Yu, Hao-Wei Zhang, Han Xiao, Jian-Hua Liu, Jian Zhang, Ke Yin

**Affiliations:** 1 Department of Orthopedics, The First Affiliated Hospital of University of South China, Hengyang, 421001 Hunan Province PR China; 2 Department of Orthopedics, Xiangya Hospital, Central South University, No. 87 Xiangya Road, Changsha, 410008 Hunan Province PR China; 3 Medical College of University of South China, Hengyang, 421001 Hunan Province PR China; 4 Department of Hematology, The First Affiliated Hospital of University of South China, Hengyang, 421001 Hunan Province PR China

**Keywords:** Stem cells, Diseases

## Abstract

Bone marrow-derived mesenchymal stem cells (BM-MSCs), the common progenitor cells of adipocytes and osteoblasts, have been recognized as the key mediator during bone formation. Herein, our study aim to investigate molecular mechanisms underlying circular RNA (circRNA) AFF4 (circ_AFF4)-regulated BM-MSCs osteogenesis. BM-MSCs were characterized by FACS, ARS, and ALP staining. Expression patterns of circ_AFF4, miR-135a-5p, FNDC5/Irisin, SMAD1/5, and osteogenesis markers, including ALP, BMP4, RUNX2, Spp1, and Colla1 were detected by qRT-PCR, western blot, or immunofluorescence staining, respectively. Interactions between circ_AFF4 and miR-135a-5p, FNDC5, and miR-135a-5p were analyzed using web tools including TargetScan, miRanda, and miRDB, and further confirmed by luciferase reporter assay and RNA pull-down. Complex formation between Irisin and Integrin αV was verified by Co-immunoprecipitation. To further verify the functional role of circ_AFF4 in vivo during bone formation, we conducted animal experiments harboring circ_AFF4 knockdown, and born samples were evaluated by immunohistochemistry, hematoxylin and eosin, and Masson staining. Circ_AFF4 was upregulated upon osteogenic differentiation induction in BM-MSCs, and miR-135a-5p expression declined as differentiation proceeds. Circ_AFF4 knockdown significantly inhibited osteogenesis potential in BM-MSCs. Circ_AFF4 stimulated FNDC5/Irisin expression through complementary binding to its downstream target molecule miR-135a-5p. Irisin formed an intermolecular complex with Integrin αV and activated the SMAD1/5 pathway during osteogenic differentiation. Our work revealed that circ_AFF4, acting as a sponge of miR-135a-5p, triggers the promotion of FNDC5/Irisin via activating the SMAD1/5 pathway to induce osteogenic differentiation in BM-MSCs. These findings gained a deeper insight into the circRNA-miRNA regulatory system in the bone marrow microenvironment and may improve our understanding of bone formation-related diseases at physiological and pathological levels.

## Introduction

Bone formation, also termed as osteogenesis or ossification, is a well-orchestrated dynamic process that is executed by osteoblasts^1^. Various human bone disorders including osteoporosis and heterotopic ossification may occur when the balance between new bone formation and old bone resorption which is regulated by osteoclasts is disrupted, or the changes of osteoblasts and adipocytes within the human bone marrow is physiological uncoordinated^2–4^. Thus, bone marrow mesenchymal stem cells (BM-MSCs), the common progenitor cells of adipocytes and osteoblasts^5^, gained recently growing attention for their function in dysregulated bone regeneration and potential application in clinical treatment for skeletal diseases^6^. The disrupted balance between osteogenesis and adipogenesis in BM-MSCs which increases fat deposits in bone marrow may promote osteoclast proliferation and contribute to subsequent bone loss. Although risk factors including aging, nutrition, corticosteroid medication usage in high doses, and an inactive lifestyle are recognized as main contributors to these bone-related disorders, underlying molecular mechanisms for the BM-MSCs lineage commitment during the progression of the variety of bone diseases are highly diverse and not well clarified so far.

Irisin is a 112-amino acid myokine which is a proteolytic cleavage product from its precursor FNDC5 (fibronectin type III domain-containing protein 5) as the extracellular receptor ectodomain. Irisin is a pleiotropic molecule and was demonstrated to be involved in thermogenesis^7^, chronic inflammation^8^, and carcinogenesis^9^. It was also reported to stimulate osteoblast differentiation and increase cortical bone strength by activating mitogen-activated protein kinase signaling pathway^10,11^. Despite its considerable roles in bone metabolism, detailed analysis is crucial to explain how Irisin exactly regulated osteogenic differentiation through recruitment of upstream and downstream signaling pathways.

Our group has been interested in the AF4/FMR2 family which regulates gene transcription through elongation and chromatin remodeling from the perspective of epigenetics. Both AFF1 and AFF4 contain three conserved domains: an N-terminal homology domain, an AF4/lymphnucleoprotein domain, and a C-terminal homology domain, and they have been shown to play important roles in HIV transcription^12,13^. Interestingly, Zhou et al.^14^ reported the differential regulation of AFF1 and AFF4 on the osteogenic differentiation in human mesenchymal stem cells and suggested their implication in bone development. Circular RNAs (circRNAs) are a type of special yet abundant endogenous noncoding RNAs that are generated by the back-splicing process and their 3′ and 5′ ends are covalently linked^15^. The application of circRNAs in disease diagnosis and treatment attracted growing attention in the last decade due to their stability and tissue-specific expression. Notably, circ_AFF4 was reported to act as a miR-7223-5p sponge and promote osteoblast proliferation by targeting miR-7223-5p/PIK3R1 axis^16^. However, it remains imperative to investigate whether circ_AFF4 mediated osteogenic differentiation process as well. Moreover, it remains elusive whether different circ_AFF4-miRNA-mRNA axis might exist under our specific pathological conditions in BM-MSCs. Further studies are required to examine the basic molecular mechanisms of the circ_AFF4-miRNA-mRNA regulatory network during bone regeneration.

In the present study, we recorded that circ_AFF4 was enhanced upon osteogenic differentiation induction, of which knockdown significantly inhibited the osteogenesis potential in BM-MSCs. By contrast, miR-135a-5p expression declined as differentiation proceeds. Further, circ_AFF4 promoted FNDC5/Irisin expression by complementary binding to miR-135a-5p, whereas Irisin formed an intermolecular complex with Integrin αV and modulated the osteogenic differentiation through the SMAD1/5 pathway. Our results shed light on the novel function of circ_AFF4 during osteogenic differentiation in BM-MSCs and suggested its potential application as a new therapeutic target in bone formation-related diseases.

## Results

### Circ_AFF4 and miR-135a-5p were differentially expressed in BM-MSCs upon osteoinduction

**Fig. 1:**
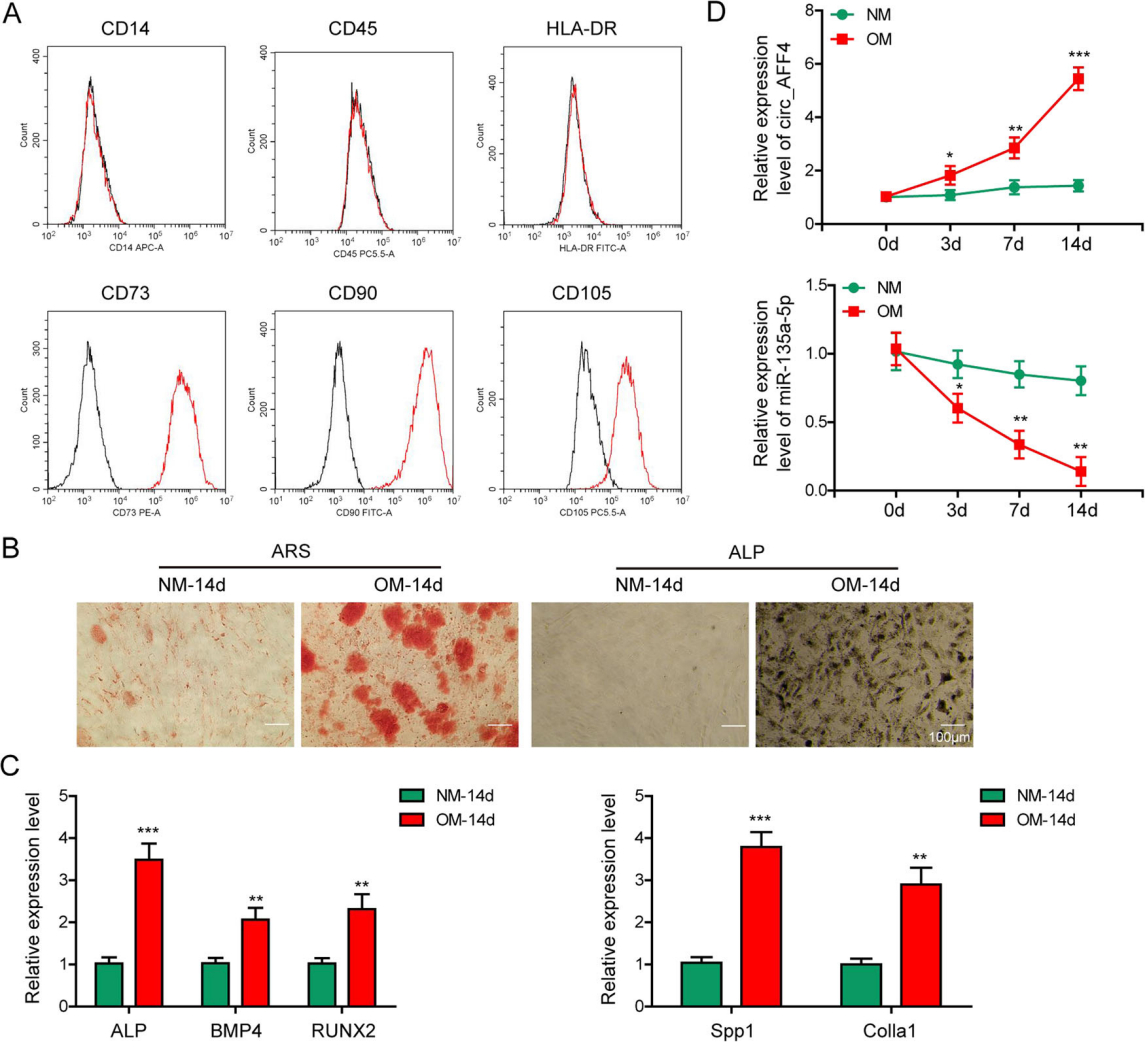
Characterization of BM-MSCs and expression profiles of circ_AFF4, miR-135a-5p during osteogenic differentiation. **A** Expression of cell surface markers BM-MSCs was analyzed by FACS. Antibodies against CD14, CD45, HLA-DR, CD73, CD90, and CD105 were used to identify BM-MSCs. **B** ARS or ALP staining of BM-MSCs showed that osteogenic differentiation was efficiently induced by using OM instead of NM. **C** qRT-PCR assay was performed to measure the mRNA expression of osteogenic marker genes ALP, BMP4, RUNX2, Spp1, and Colla1. **D** BM-MSCs were cultured in osteogenic medium (OM) or normal medium (NM). Relative expression of circ_AFF4 and miR-135a-5p was detected by qRT-PCR at days 0, 3, 7, and 14. The data were shown as mean ± SD based on at least three independent experiments. *P < 0.05, **P < 0.01, and ***P < 0.001.

In order to examine the biological roles of circ_AFF4 and miR-135a-5p during osteogenic differentiation, we purchased human BM-MSCs from Lonza. w. As defined by the mesenchymal and tissue stem cell committee of the International Society for Cellular Therapy (ISCT), expression of surface markers CD73, CD90, CD105, and lack expression of surface markers CD45, CD34, and CD14/CD19 and HLA-DR of are used to identify MSCs. In consistence with this criterion, our FACS analysis for the expression of the surface markers identified the isolated BM-MSCs (Fig. 1A). We next cultured BM-MSCs in osteogenic medium (OM) for osteogenic differentiation induction and evaluated their differential potential by conducting ARS and ALP staining (Fig. 1B). As for the multi-differentiation capacity of BM-MSCs, we observed increasing lipid accumulation, and lipid droplets gradually became larger, which suggested its adipogenic differentiation potential (Fig. S1). Alcian blue staining also showed the potential of BM-MSCs to differentiate into chondrocytes (Fig. S1). BM-MSCs treated with OM displayed positive staining with both ARS and ALP and demonstrated efficient osteogenic differentiation induction in comparison to those grown in a normal medium (NM). We also compared expression levels of osteogenesis markers, including ALP, BMP4, RUNX2, Spp1, and Colla1. Our quantitative real-time polymerase chain reaction (qRT-PCR) results showed that these osteogenesis-related genes were greatly upregulated in OM-treated BM-MSCs as compared to those grown in NM (Fig. 1C), further confirming the osteoinduction effect in OM-treated BM-MSCs. We performed qRT-PCR analysis and observed that circ_AFF4 was markedly upregulated upon osteogenic differentiation induction, whereas miR-135a-5p was down-regulated in BM-MSCs as differentiation proceeded (Fig. 1D). These results demonstrated the successful characterization of BM-MSCs and revealed the altered expression profiles of circ_AFF4 and miR-135a-5p during osteogenic differentiation.

### Circ_AFF4 knockdown suppressed BM-MSCs osteogenic differentiation

**Fig. 2:**
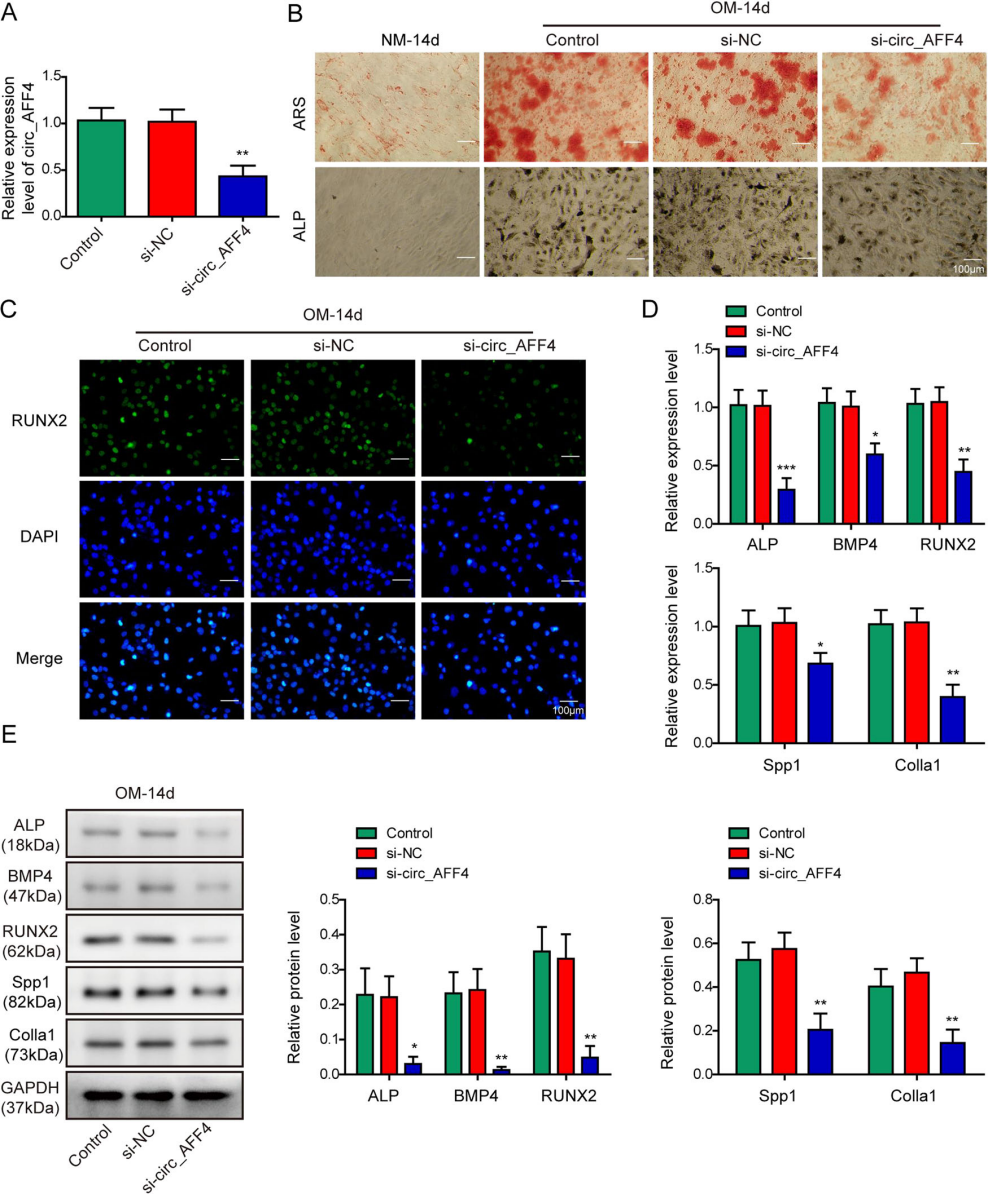
Silencing of circ_AFF4 attenuated the osteogenic differentiation in BM-MSCs. **A** BM-MSCs were transfected with si-circ_AFF4 or the negative control. Relative expression of circ_AFF4 was detected by qRT-PCR. **B** BM-MSCs transfected with si-circ_AFF4 or the negative control were further cultured in OM or NM for 14 days. Osteogenic differentiation was evaluated by performing ARS and ALP staining assays. **C** RUNX2 expression was determined by immunofluorescence staining utilizing RUNX2-specific antibodies. **D** Fourteen days after the osteogenic differentiation induction, qRT-PCR was performed to measure the mRNA expression of osteogenic marker genes ALP, BMP4, RUNX2, Spp1, and Colla1 in BM-MSCs. **E** Protein expression of ALP, BMP4, RUNX2, Spp1, and Colla1 was determined by western blot analysis. The data were shown as mean ± SD based on at least three independent experiments. *P < 0.05, **P < 0.01, and ***P < 0.001.

Before answering the question whether circ_AFF4 participated as a regulatory molecule during osteogenic differentiation processes in BM-MSCs, we first characterized circ_AFF4 by agarose gel electrophoresis, of which presence was proved by qRT-PCR (Fig. S2A). In addition, the data of RNase R assay showed that the expression of linear form of AFF4 in BM-MSCs was significantly decreased under the RNase R treatment, while circ_AFF4 resist to RNase R digestion (Fig. S2B). To further validate the role of circ_AFF4, we then designed three siRNAs that specifically target circ_AFF4 and selected one of them with highest knockdown efficiency by performing qRT-PCR assay (Fig. S2C). We transfected BM-MSCs with si-circ_AFF4 and qRT-PCR assay showed that circ_AFF4 expression was notably decreased in comparison to negative control (Fig. 2A), but no impact was observed for the expression of linear AFF4 mRNA ((Fig. S2D). Next, we maintained the BM-MSCs above in OM for 14 days and assessed the in vitro osteogenic differentiation potential by performing ARS and ALP staining. Interestingly, BM-MSCs with circ_AFF4 knockdown exhibited greatly lower ARS and ALP intensity than in negative control, indicating the reduced osteogenic differentiation capacity (Fig. 2B). We then cultured the BM-MSCs above in OM for 3 days and analyzed the RUNX2 expression by immunofluorescence (IF) analysis. The number of RUNX2-positive cells has obviously declined in si-circ_AFF4-treated BM-MSCs than in negative controls (Fig. 2C). Furthermore, we analyzed expression levels of osteogenesis markers including ALP, BMP4, RUNX2, Spp1, and Colla1, and observed that osteogenesis-related genes were effectively inhibited at both mRNA and protein levels by silencing of circ_AFF4 (Fig. 2D, E). These data implied that circ_AFF4 might exert a positive role in regulating the capacity of osteogenic differentiation in BM-MSCs.

### miR-135a-5p was a downstream target to participate in circ_AFF4-mediated BM-MSCs osteogenic differentiation

**Fig. 3:**
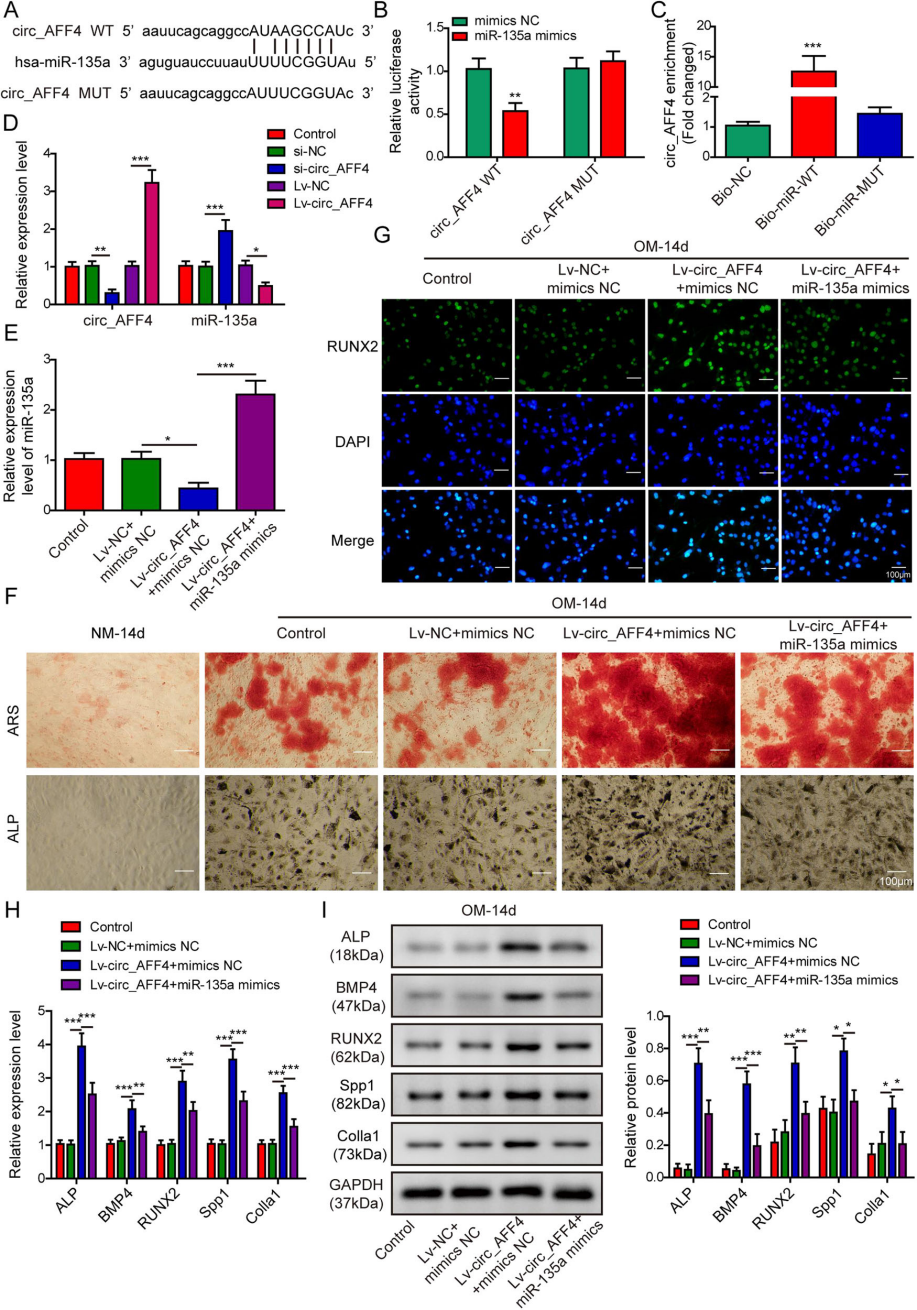
miR-135a-5p modulated the biological effects of circ_AFF4 during osteogenic differentiation by functioning as its downstream target gene. **A** Potential targeted binding between miR-135a-5p and AFF4 was predicted by using TargetScan and Starbase web tools. The identified complementary binding sites were further validated by performing dual-luciferase reporter (**B**) and RNA pull-down assays (**C**). **D** BM-MSCs were transfected with lentiviruses encoding circ_AFF4 or empty viruses, si-circ_AFF4 or si-NC. Relative expression of miR-135a-5p and circ_AFF4 was detected by qRT-PCR. **E** BM-MSCs were co-transfected with lentiviruses encoding circ_AFF4 and miR-135a-5p mimics or miRNA negative control. The relative expression of miR-135a-5p was measured by qRT-PCR. **F** BM-MSCs were co-transfected with lentiviruses encoding circ_AFF4 and miR-135a-5p mimics or miRNA negative control, and further cultured in OM or NM for 14 days. ARS and ALP staining were performed to evaluate the osteogenic differentiation in BM-MSCs. **G** BM-MSCs were co-transfected with lentiviruses encoding circ_AFF4 and miR-135a-5p mimics or miRNA negative control, and further cultured in OM for 14 days. RUNX2 expression was analyzed by immunofluorescence staining using RUNX2-specific antibodies. **H** Fourteen days after osteogenic differentiation induction, the qRT-PCR assay was performed to determine the mRNA expression of osteogenic marker genes ALP, BMP4, RUNX2, Spp1, and Colla1 in BM-MSCs. **I** Protein expression levels of the osteogenic marker genes above was determined using western blot. The data were shown as mean ± SD based on at least three independent experiments. *P < 0.05, **P < 0.01, and ***P < 0.001.

Next, we conducted bioinformatics analysis and identified miR-135a-5p as the potential target molecule for circ_AFF4. The computationally predicted binding sites between miR-135a-5p and circ_AFF4 were shown in Fig. 3A and validated experimentally by luciferase reporter assay. As shown in Fig. 3B, relative luciferase activity of circ_AFF4-WT was largely reduced upon co-transfection of miR-135a-5p mimics as compared to miR-NC. However, luciferase activity of circ_AFF4-MUT harboring mutations in miR-135a-5p binding sites remained completely unchanged in the presence of miR-135a-5p mimics or miR-NC. The intermolecular interactions between circ_AFF4 and miR-135a-5p were further tested and confirmed by RNA pull-down assay Enriched circ_AFF4-WT but not circ_AFF4-MUT was detected by using a biotinylated probe that was specifically against miR-135a-5p (Fig. 3C). To thoroughly understand the regulatory effect of circ_AFF4 on miR-135a-5p during osteogenic differentiation, we first transfected BM-MSCs with lentiviruses encoding circ_AFF4 for stable overexpression and si-circ_AFF4 for a stable knockdown. Intriguingly, miR-135a-5p expression was greatly inhibited by circ_AFF4 overexpression and promoted by circ_AFF4 knockdown (Fig. 3D). However, co-transfection with Lv-circ_AFF4 and miR-135a-5p mimics restored and effectively increased miR-135a-5p levels in BM-MSCs (Fig. 3E). Next, we evaluated the interactive impact of circ_AFF4 and miR-135a-5p on BM-MSC’s osteogenic differentiation capacity. As illustrated in Fig. 3F, BM-MSCs co-transfected with circ_AFF4 and miR-135a-5p mimics displayed attenuated staining intensity of both ALP and ARS as compared to those transfected with circ_AFF4 and miR-NC, suggesting that the promotion of osteogenic differentiation by circ_AFF4 was partial, if not completely, abolished by miR-135a-5p. Consistently, we noted that miR-135a-5p clearly repressed RUNX2 protein expression as differentiation proceeds, which was directly induced by circ_AFF4 upregulation (Fig. 3G). Furthermore, quantification of the osteogenesis-related genes including ALP, BMP4, RUNX2, Spp1, and Colla1 revealed up to 40% expression reduction of all these osteogenic markers analyzed at mRNA (Fig. 3H) and protein (Fig. 3I) levels. Meanwhile, co-localization of circ_AFF4 and miR-135a-5p in the cytoplasm of BM-MSCs was verified by fluorescence in situ hybridization (FISH) assay (Fig. S3A). In addition, the RIP assay demonstrated that circ_AFF4 was enriched in Ago-containing immune-precipitates (Fig. S3B). Our data provided the first evidence that circ_AFF4 might modulate osteogenic differentiation and this impact was exerted through interacting with miR-135a-5p.

### Circ_AFF4 upregulated FNDC5/Irisin through direct binding of miR-135a-5p

**Fig. 4:**
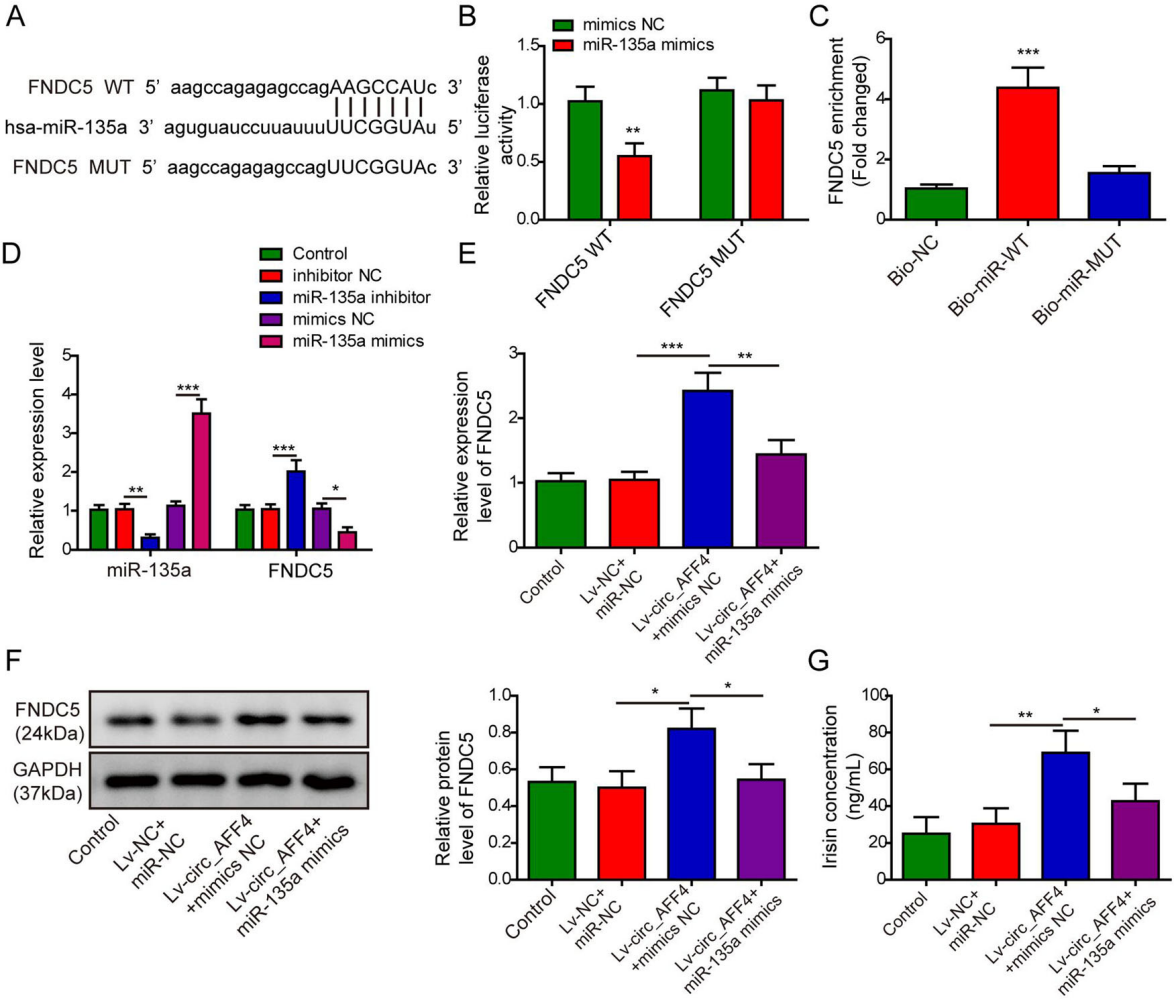
circ_AFF4 enhanced expression of FNDC5/Irisin through targeted binding with miR-135a-5p. **A** Potential binding sites between FNDC5 and miR-135a-5p were predicted by using the RNAInter web tool. The complementary binding between FNDC5 and miR-135a-5p was validated by dual-luciferase reporter (**B**) and RNA pull-down assays (**C**). **D** BM-MSCs were transfected with lentiviruses expressing miR-135a-5p mimics, miR-135a-5p inhibitor, or negative control. Relative expression of miR-135a-5p and FNDC5 was determined using qRT-PCR. **E** BM-MSCs were co-transfected with lentiviruses encoding circ_AFF4 and miR-135a-5p mimics or miRNA negative control. Molecular expression of FNDC5 at mRNA and protein levels was analyzed by qRT-PCR (**E**) and western blot (**F**) analysis respectively. **G** Irisin expression in BM-MSCs was quantified by ELISA using an anti-Irisin antibody. The data were shown as mean ± SD based on at least three independent experiments. *P < 0.05, **P < 0.01, and ***P < 0.001.

To predict the potential downstream binding target of miR-135a-5p, we implemented RNAInter database analysis and identified 7 nucleotides base-pairing between miR-135a-5p and FNDC5 (Fig. 4A). A luciferase reporter assay was conducted to validate the computed binding sites above. The luminescence intensity of FNDC5-WT was significantly reduced upon co-transfection of miR-135a-5p mimics as compared to miR-NC. By contrast, luciferase activity of FNDC5-MUT harboring mutations in miR-135a-5p binding sites remained approximately the same in the presence of miR-135a-5p mimics or miR-NC (Fig. 4B). Intermolecular interactions between miR-135a-5p and FNDC5-WT, but not FNDC5-MUT were further confirmed by RNA pull-down assay using biotinylated probe specifically against miR-135a-5p (Fig. 4C). To investigate the regulatory effect of miR-135a-5p on FNDC5 during osteogenic differentiation, we transfected BM-MSCs with miR-135a-5p mimics for overexpression and miR-135a-5p inhibitors for a knockdown. qRT-PCR analysis showed that FNDC5 expression was increased by miR-135a-5p inhibition but repressed by miR-135a-5p overexpression (Fig. 4D). Interestingly, we discovered the enhanced FNDC5 mRNA levels in circ_AFF4-overexpressing BM-MSCs. However, the modest upregulation was completely abrogated by co-transfection of miR-135a-5p mimics (Fig. 4E). Consistently, BM-MSCs transfected Lv-circ_AFF4 exhibited elevated protein expression of FNDC5, and the effect of promotion was abolished by miR-135a-5p overexpression (Fig. 4F). Analogous to the results with FNDC5, we assessed Irisin expression in BM-MSCs by performing ELISA and observed that Irisin concentration was increased by circ_AFF4 and mostly reversed by co-transfection with miR-135a-5p mimics (Fig. 4G). Taken together, our findings above demonstrated for the first time that circ_AFF4 acted as a sponge of miR-135a-5p to promote the expression of FNDC5/Irisin in osteogenic differentiation.

### Promotion of circ_AFF4 on osteogenic differentiation was counteracted by FNDC5/Irisin downregulation

**Fig. 5:**
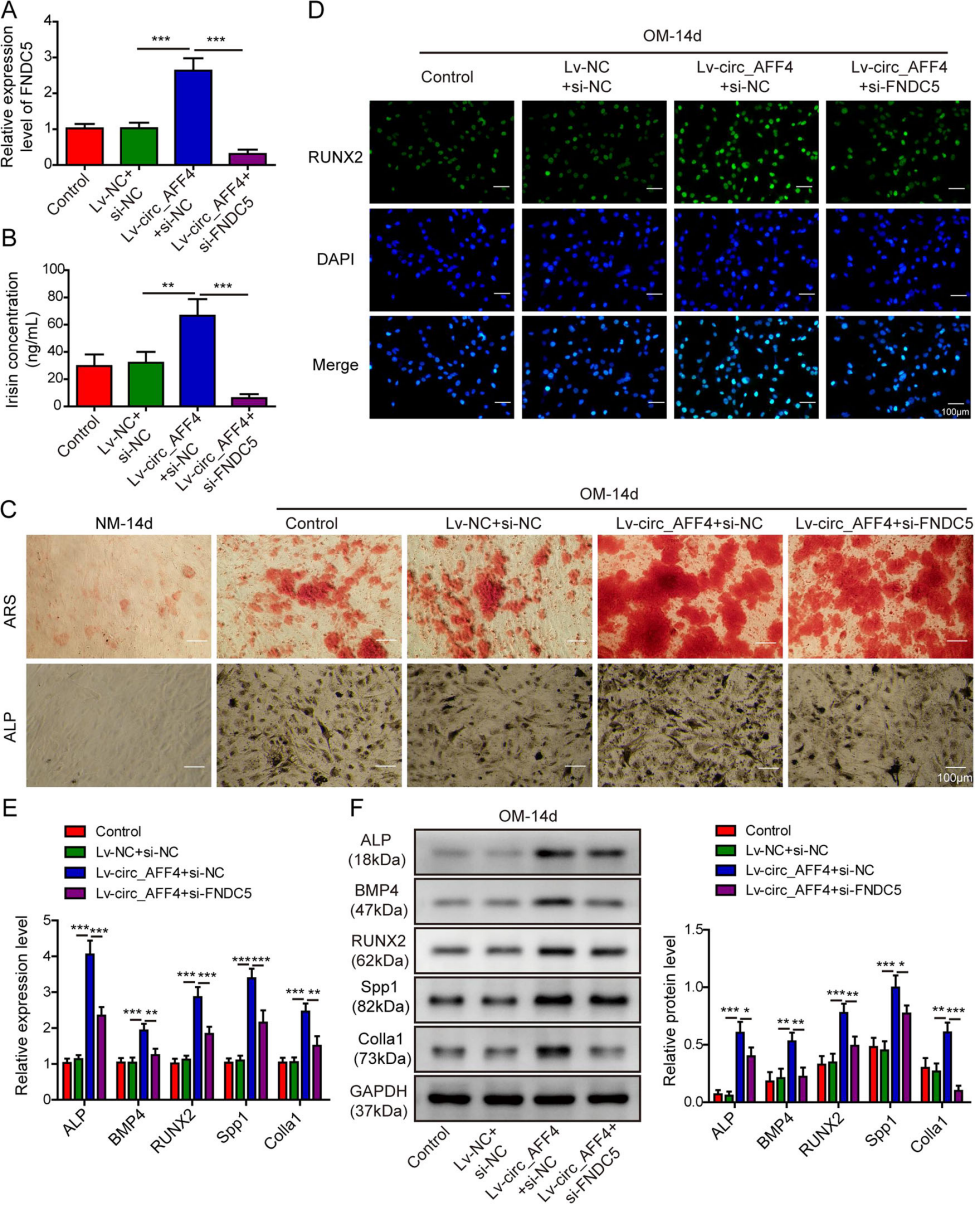
Promotion of circ_AFF4 on osteogenic differentiation was partially reversed by silencing of FNDC5/Irisin. BM-MSCs were co-transfected with lentiviruses expressing circ_AFF4 and si-FNDC5 or negative control siRNA. **A** Relative expression of FNDC5 was detected by qRT-PCR. **B** Irisin concentration was determined by ELISA. **C** Transfected BM-MSCs were further cultured in OM or NM for 14 days. ARS and ALP staining assays were performed to analyze the osteogenic differentiation. **D** Transfected BM-MSCs were further cultured in OM for 14 days. RUNX2 expression was detected by immunofluorescence staining using RUNX2-specific antibodies. qRT-PCR and western blot analyses were performed to evaluate the mRNA (**E**) and protein (**F**) expression of osteogenic marker genes ALP, BMP4, RUNX2, Spp1, and Colla1 in BM-MSCs. The data were shown as mean ± SD based on at least three independent experiments. *P < 0.05, **P < 0.01, and ***P < 0.001.

To investigate the regulatory relationship between circ_AFF4 and FNDC5/Irisin in BM-MSCs, we transfected the latter with recombinant lentiviruses harboring circ_AFF4 and si-FNDC5 for stable FNDC5 knockdown or si-NC as a negative control. Quantification of FNDC5 by qRT-PCR (Fig. 5A) and Irisin by ELISA (Fig. 5B) showed that si-FNDC5 treatment effectively repressed FNDC5 mRNA expression as well as Irisin peptide accumulation in differentiating BM-MSCs, which was promoted by circ_AFF4 overexpression. To validate our speculation whether and how circ_AFF4 and FNDC5/Irisin interactively regulated osteogenic differentiation, we cultured BM-MSCs which were transfected with circ_AFF4 and si-FNDC5 or si-NC in OM for 14 days and evaluated the osteogenesis capacity by ARS and ALP staining. As shown in Fig. 5C, FNDC5 knockdown via RNA interference clearly attenuated ARS and ALP intensity which was enhanced by circ_AFF4 overexpression. Consistently, we observed that the number of RUNX2 positive BM-MSCs, which was enlarged upon circ_AFF4 treatment, was significantly decreased by the simultaneous silencing of FNDC5 (Fig. 5D). We also assessed the expression of osteogenesis-related genes including ALP, BMP4, RUNX2, Spp1, and Colla1 in BM-MSCs transfected with circ_AFF4 and si-FNDC5 or negative control. As differentiation proceeds, the expression of all these osteogenesis markers was substantially stimulated by circ_AFF4 at both mRNA (Fig. 5E) and protein levels (Fig. 5F). However, the promotion effect was moderately counteracted by silencing of FNDC5. Collectively, our results revealed that the pro-osteogenic function of circ_AFF4 was partially compromised by FNDC5/Irisin downregulation.

### Irisin bound Integrin αV and mediated osteogenic differentiation of BM-MSCs via activation of SMAD1/5 pathway

**Fig. 6:**
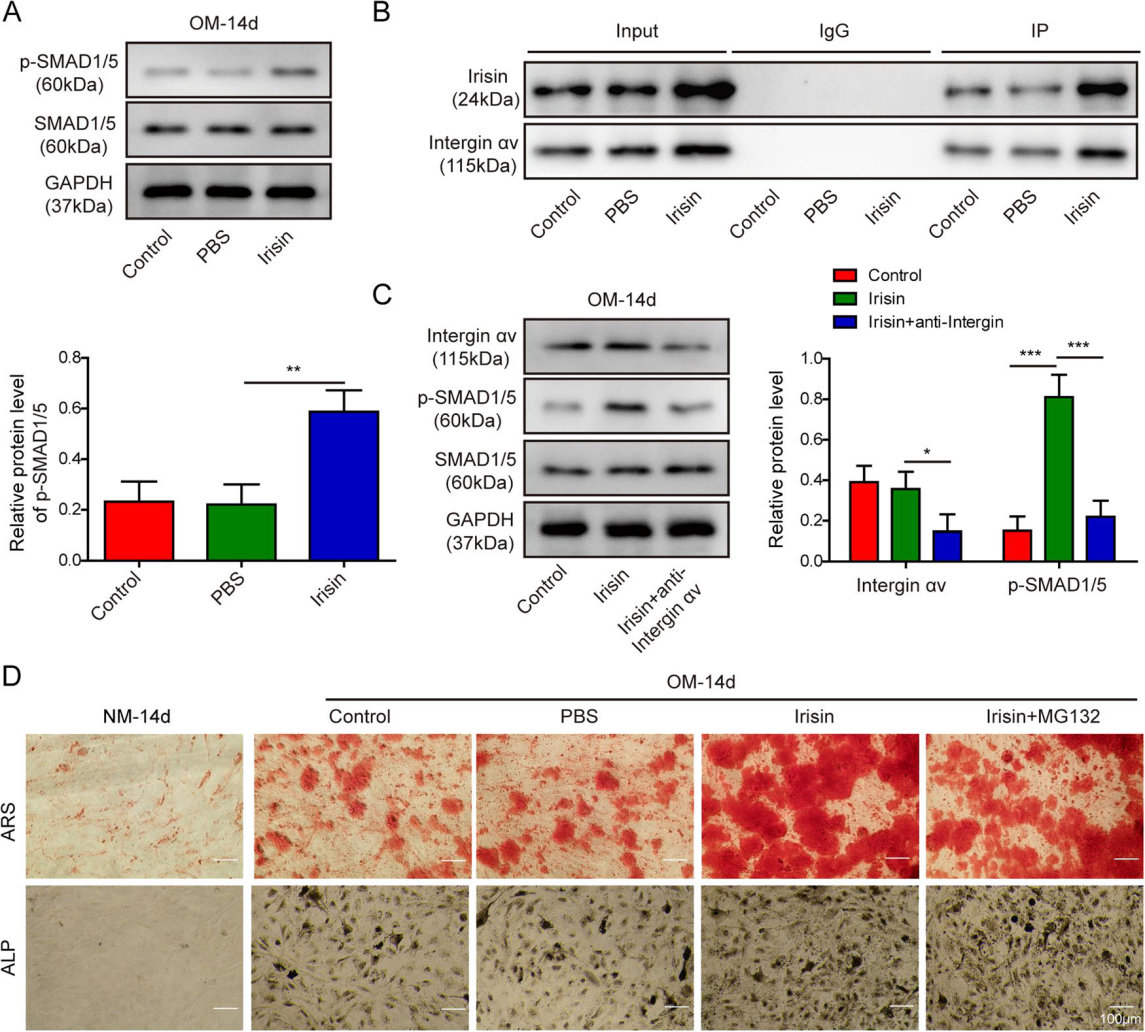
Irisin-mediated osteogenic differentiation in BM-MSCs through specific binding with Integrin αV and activation of the SMAD1/5 pathway. **A** BM-MSCs were transfected with pcDNA3.1 vector containing Irisin and further cultured in OM for 14 days. WB was performed to analyze the expression of SMAD1/5 and phosphorylated SMAD1/5. **B** Intermolecular interaction between Irisin and the transmembrane receptor Integrin αV was tested by Co-IP. **C** BM-MSCs were transfected with Irisin-overexpressing pcDNA3.1 vector alone and further cultured in OM containing Integrin αV specific antibody for 14 days. WB was performed to measure the expression of Integrin αV, SMAD1/5, and phosphorylated SMAD1/5. **D** ARS and ALP staining assays were performed to evaluate the osteogenic differentiation in Irisin-overexpressing BM-MSCs and those treated with MG132. The data were shown as mean ± SD based on at least three independent experiments. *P < 0.05, **P < 0.01, and ***P < 0.001.

A recent study showed that Integrin αV functioned as an Irisin receptor in osteocytes and fat tissues and contributed to the bone remodeling regulation^17^. To explore the possible underlying mechanisms of the Irisin/Integrin αV complex during osteogenic and adipogenic differentiation and answer the question of whether Irisin is an upstream regulator of SMAD1/5, we transfected BM-MSCs which were maintained in OM for 14 days with pcDNA3.1 vector encoding Irisin. WB analysis showed that Irisin overexpression stimulated the expression of phosphorylated SMAD1/5 (p-SMAD1/5); however, the expression level of SMAD1/5 in inactive unphosphorylated conformation remained unaltered in comparison to the negative controls (Fig. 6A). Next, we applied Co-IP to test whether Irisin and Integrin αV physiologically binds each other in BM-MSCs. As illustrated in Fig. 6B, Integrin αV was highly selectively enriched in Irisin-overexpressing BM-MSCs, as compared to the negative controls, and substantiated the in vitro complex formation between Irisin and Integrin αV. We also examined whether Integrin αV was implicated in SMAD1/5 pathway regulation. Indeed, downregulation of Integrin αV using Integrin αV inhibitor obviously diminished the p-SMAD1/5 molecular expression level which was stimulated by Irisin overexpression and resulted in the partial blockage of a SMAD1/5 signaling pathway (Fig. 6C). Previous studies showed that the activation of SMAD1/5 was indispensable for osteogenesis differentiation^18,19^. In accordance, we found that deactivation of SMAD1/5 pathway using the cell-permeable proteasome inhibitor MG132 effectively suppressed osteogenesis differentiation which was induced by Irisin, as visualized by ALP and ARS staining (Fig. 6D).

### The pro-osteogenic function of circ_AFF4 depended on the SMAD1/5 pathway

**Fig. 7:**
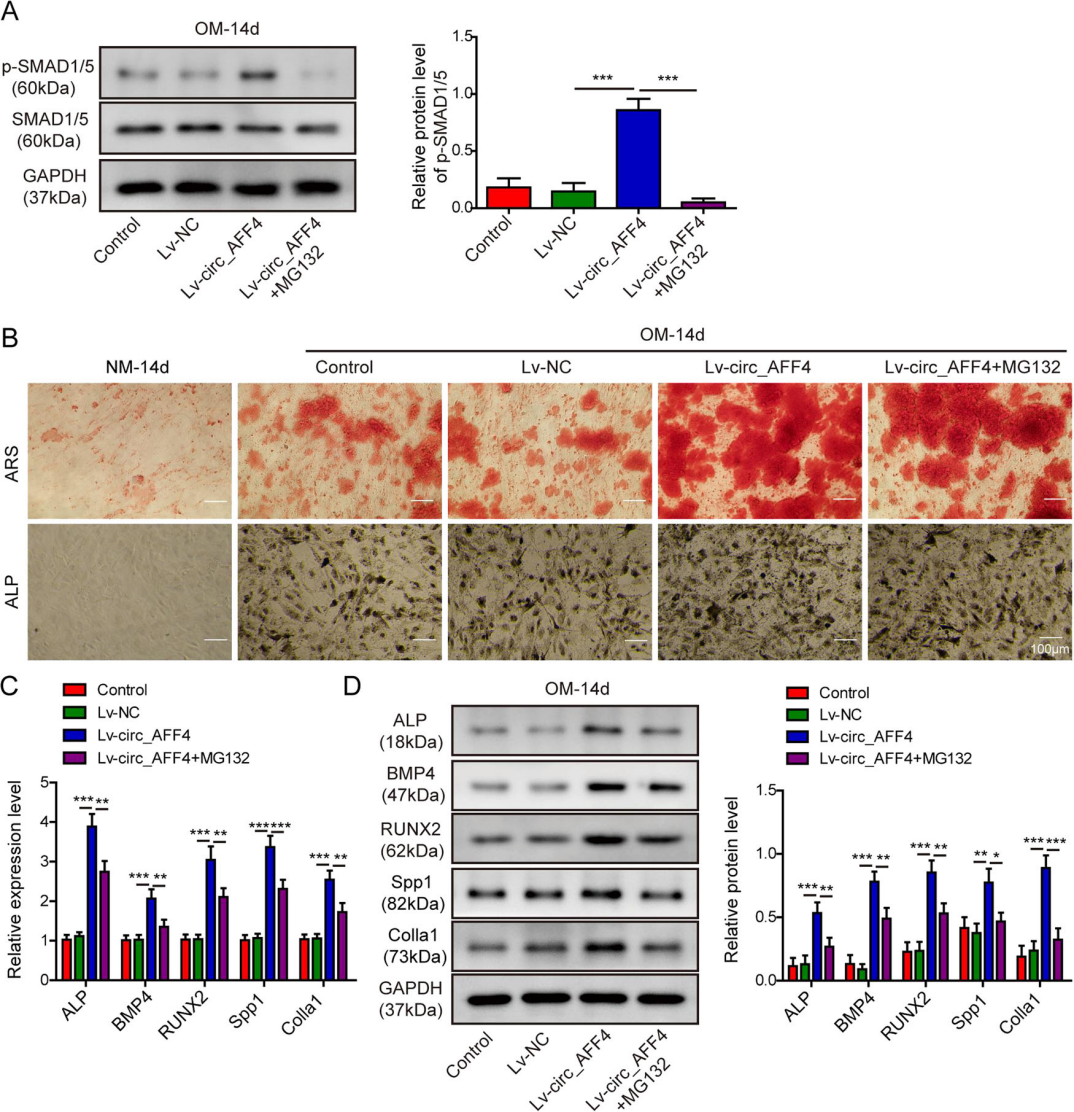
Promotion of circ_AFF4 on osteogenic differentiation in BM-MSCs is associated with the SMAD1/5 pathway. BM-MSCs were transfected with lentiviruses encoding circ_AFF4 alone or co-treated with MG132 and further cultured in OM for another 14 days. **A** Expression of SMAD1/5 and phosphorylated SMAD1/5 was detected by WB analysis. **B** Osteogenic differentiation in BM-MSCs was evaluated by ARS and ALP staining assays. Expression of osteogenic marker genes ALP, BMP4, RUNX2, Spp1, and Colla1 in BM-MSCs was determined by qRT-PCR (**C**) and western blot (**D**). The data were shown as mean ± SD based on at least three independent experiments. *P < 0.05, **P < 0.01, and ***P < 0.001.

Thus far, we have demonstrated the up-regulatory effect of circ_AFF4 on Irisin expression in OM-induced BM-MSCs and depicted that Irisin mediated osteogenesis differentiation by activating SMAD1/5. We next attempted to address the question of whether the pro-osteogenic function of circ_AFF4 was also associated with the SMAD1/5 pathway. As shown in Fig. 7A, we detected enhanced accumulation of p-SMAD1/5 in BM-MSCs which were transfected with recombinant lentiviruses encoding circ_AFF4 and cultured in OM for 14 days. However, p-SMAD1/5 expression markedly diminished after the cells were treated with MG132. By contrast, the molecular level of unphosphorylated SMAD1/5 remained unchanged, suggesting that the SMAD1/5 pathway was activated by circ_AFF4 overexpression. We next evaluated whether osteogenesis differentiation processes in BM-MSCs were affected by SMAD1/5 deactivation. As speculated, we observed that the staining intensity of ARS and ALP, which was increased by circ_AFF4 upregulation, clearly declined upon MG132 treatment (Fig. 7B), indicating the reduced osteogenesis differentiation capacity of BM-MSCs. Moreover, inhibition of the SMAD1/5 pathway with MG132 abrogated the stimulating effect of circ_AFF4 on the osteogenesis markers including ALP, BMP4, RUNX2, Spp1, and Colla1, and largely suppressed their expression at both mRNA (Fig. 7C) and protein levels (Fig. 7D). These results substantiated our hypothesis that the regulatory function of circ_AFF4 on the osteogenic differentiation in BM-MSCs was partially exerted through the SMAD1/5 pathway.

### In vivo suppression of bone formation by circ_AFF4 knockdown

**Fig. 8:**
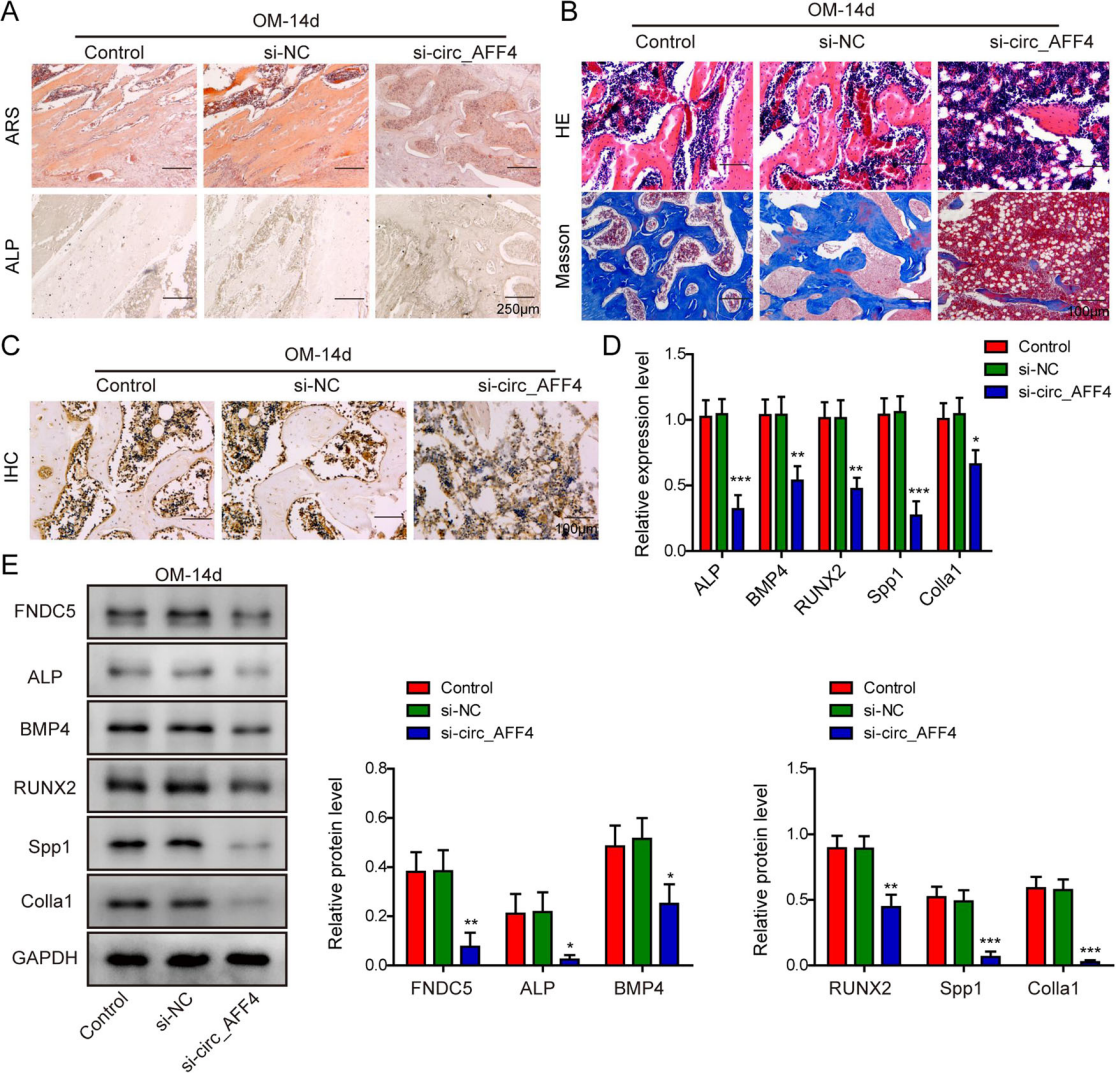
In vivo bone formation was inhibited by circ_AFF4 knockdown. BM-MSCs were transfected with si-circ_AFF4 or siRNA negative control (NC) and further cultured in OM for 7 days. BALB/c nude mice were injected with the BM-MSCs above and femurs were removed after 8 weeks. **A** ARS and ALP staining assays were performed to compare the bone cell formation in mice treated with si-circ_AFF4 or si-NC. **B** Representative images of H&E and Masson staining in mouse femurs showing the in vivo bone formation and collagen deposition, respectively. **C** Expression of the osteogenic differentiation-related transfection factor RUNX2 was evaluated using IHC staining. qRT-PCR (**D**) and WB (**E**) were conducted to determine the expression levels of osteogenic marker genes ALP, BMP4, RUNX2, Spp1, and Colla2 in femur samples from mice treated with si-circ_AFF4 or siRNA NC. The data were shown as mean ± SD based on at least three independent experiments. *P < 0.05, **P < 0.01, and ***P < 0.001.

To explore the in vivo role of circ_AFF4 in bone formation, we loaded BM-MSCs which were transfected with circ_AFF4 siRNA or its negative control on scaffold material circ_AFF4 and implanted in mice. As visualized by ALP and ARS staining, bone samples from mice with circ_AFF4 knockdown displayed diminished bone cell differentiation and calcium deposits (Fig. 8A). We also evaluated the new bone formation with other histological staining approaches. H&E staining revealed significantly reduced new bone regeneration in si-circ_AFF4 treated mice in comparison to the control groups (Fig. 8B). Analogously, Masson’s trichrome staining showed that silencing of circ_AFF4 decreased the collagen deposition at the new bone formation zone and hindered the new bone growth consequently (Fig. 8B). Next, we assessed RUNX2 expression by performing IHC staining. As shown in Fig. 8C, RUNX2 protein synthesis was significantly suppressed by silencing of circ_AFF4. Furthermore, qRT-PCR and western blot analysis of the bone tissue samples demonstrated that molecular expression of the osteogenesis markers ALP, BMP4, RUNX2, Spp1, and Colla1 was much lower from mice implanted with si-circ_AFF4 scaffolds at both mRNA (Fig. 8D) and protein levels (Fig. 8E) than those implanted with negative controls. Consistently, the effect of circ_AFF4 overexpression on promoting osteogenesis in vivo was also verified (Fig. S4A–D). Collectively, these data together provided the first in vivo experimental evidence that bone regeneration was largely inhibited by circ_AFF4 downregulation, but promoted by circ_AFF4 overexpression.

## Discussion

As the pluripotent progenitor cells that can self-renew and differentiate into multiple cell lineages including osteoblasts, chondroblasts, neural cells, and adipocytes, BM-MSCs are an essential bone-regeneration regulator at physiological and pathological conditions. Cell lineage commitment is often aberrantly shifted in diseased microenvironments and patients suffer from progressive bone loss which is accompanied by marrow fat accumulation^20^. Although the therapeutic application of BM-MSCs represents an appealing strategy for skeletal disease treatment and already showed some promising effects in certain clinical practices, i.e., large bone fracture healing and bone tissue engineering^21,22^, further investigation is required to clarify the regulatory mechanism of BM-MSCs osteoblast differentiation and provide the theoretical guide for future therapeutic interventions. In the current study, we have revealed for the first time that BM-MSCs osteogenesis was coordinately controlled by a novel molecular network consisting of circ_AFF4, miR-135a-5p, FNDC5/Irisin, Integrin αV, and SMAD1/5. We also demonstrated that bone formation was inhibited by in vivo knockdown of circ_AFF4. Nevertheless, our future work should investigate the in vivo effects of circ_AFF4 more comprehensively by employing micro-CT quantitative approaches. CircRNAs, a class of unique long noncoding RNAs, attracted lately increasing attention for their tight association with osteogenesis in BM-MSCs^23,24^ and potential implication in various bone diseases^25^. Provided that funds are sufficient, it would be important to perform the whole screening procedure and verify all the possible circRNAs during osteogenic differentiation of BM-MSCs. Our group is interested in the AF4/FMR2 family, whereas the circRNA version of AFF4 was reported to promote osteoblast proliferation by sponging the miR-7223-5p/PIK3R1 axis^16^. In accordance, we observed that circ_AFF4 was significantly upregulated during osteoblast differentiation in BM-MSCs, whereas silencing of circ_AFF4 suppressed in vitro osteogenic progression and in vivo bone formation. Our results highlighted the important role of circ_AFF4 during the osteogenesis process.

MicroRNAs (miRNAs) are another type of noncoding RNA molecules that were found to play important regulatory roles during skeletal development, and an increasing number of miRNAs, including miR-135a, was demonstrated to control osteogenic lineage progression though targeting specific transcription factors and signaling pathways (e.g., BMP, ELK, and TLR4 pathways^26–28^). In addition, Kim and colleagues reported that miR-135a overexpression resulted in tooth formation failure^29^. Herein, we detected significantly diminished expression of miR-135a-5p, the mature form of miR-135a, as osteogenesis proceeds in BM-MSCs. In addition, we verified the competitive binding between circ_AFF4 and miR-135a-5p and revealed that the promotion effect of circ_AFF4 on osteogenic differentiation was directly mediated by miR-135a-5p. The competing endogenous RNAs (ceRNA) hypothesis suggests that circRNAs influenced gene transcription, mRNA turnover and protein translation by acting as efficient miRNA sponges. CircRNAs competitively bind a specific miRNA through its miRNA response elements and represses the miRNA inhibitory activity on its target mRNA. CircRNAs may bind multiple miRNA downstream targets, therefore, there exist potentially different circRNA–miRNA–mRNA regulatory axis under certain pathological conditions. Our group is especially interested in the regulatory mechanism of Irisin in osteogenic differentiation and chose to investigate the complementary binding between its coding gene FNDC5 and miR-135a, therefore, we identified and focused on the interaction between circ_AFF4, miR-135a-5p/Fndc5/Irisin, and their downstream SMAD pathway in the present study. Although we have chosen the optimal transfection-concentrations of circ_AFF4 and miR-135a by performing preliminary experiments, we speculate that their effects on osteogenic differentiation in BM-MSCs are not dose-dependent. By contrast, cellular damages were increased with higher transfection concentration, whereas cell functions were inhibited to some extent. However, exact molecular mechanisms need to be further investigated. Future work is also needed to address the important questions including whether another circ_AFF4-miRNA axis is involved during born formation, and how these circRNA-miRNA-mRNA network components are interacting with each other. Our findings expanded our knowledge of circRNA–miRNA regulatory network in the bone marrow microenvironment and improved our understanding of fine-tuning gene expression during bone formation processes.

In summary, our work revealed the pro-osteogenic function of circ_AFF4 in BM-MSCs through direct regulation of miR-135a-5p/FNDC5/Irisin/Integrin αV/SMAD1/5 axis. By highlighting the potential value of circ_AFF4 as an appealing therapeutic target for bone formation-related diseases, our work provided a profound insight into the molecular mechanisms of osteogenic lineage regulation in BM-MSCs.

## Materials and methods

### Cultivation and characterization of BM-MSCs

Human BM-MSCs were purchased from Lonza and gently resuspended in Dulbecco’s modified Eagle’s medium (DMEM) (Gibco, Grand Island, NY, USA) which was supplemented with 10% fetal bovine serum (Gibco, Grand Island, NY, USA), 100 U/mL penicillin, and 0.1 mg/mL streptomycin (Sigma, St Louis, MO, USA). Cells were plated on a 10 cm cell culture dish at a seeding density of 1 × 10^5^ cells/cm^2^ and incubated at 37 °C in a humidified environment with 5% CO_2_. The medium was changed every 2 or 3 days and BM-MSCs from passage 3–5 were used for the subsequent experiments.

BM-MSCs were washed gently with phosphate-buffered saline (PBS) and characterized by performing flow cytometry using fluorescein isothiocyanate conjugated antibodies against CD14, CD45, and HLA-DR; phycoerythrin-conjugated antibodies against CD73, CD90; PerCP-Cy 5.5 conjugated antibody against CD105. Antibodies were purchased from BD Biosciences (San Jose, CA, USA).

### Cell transfection

The si-RNA specifically targeting the back-splice junction of circ_AFF4 (si-circ_AFF4) was designed and generated by GenePharma (Shanghai, China). Single-stranded miR-135a-5p mimics that mimic mature miR-135a-5p, a high-affinity miR-135a-5p inhibitor for a miR-135a-5p knockdown, small interfering RNAs (si-RNA) against FNDC5, and the corresponding miRNA or si-RNA control were purchased from GenePharma (Shanghai, China). These plasmids mentioned above and their corresponding negative controls were transfected using Lipofectamine 3000 (Invitrogen, Carlsbad, CA, USA), according to the manufacturer’s protocol.

As for lentivirus infection, circ_AFF4 overexpression lentivirus and vector control were purchased from GenePharma. BM-MSCs were seeded on a 6-well plate at a density of 5 × 10^4^ cells/ml and grown to 60% confluency. Cells were infected with lentiviruses in the presence of 8 μg/mL polybrene at a multiplicity of infection of 20. After incubation for 48 h at 37 °C, transduced BM-MSCs were selected using 5 μg/mL of puromycin.

### Osteogenic differentiation induction

BM-MSCs osteogenic differentiation induction was conducted using Human Mesenchymal Stem Cell (hMSC) Osteogenic Differentiation Medium BulletKit (Lonza, Switzerland) by following manufacturer instructions. Briefly, BM-MSCs were seeded on 24-well plate with a density of 1 × 10^4^ cells/well and cultured in complete DMEM at 37 °C, 5% CO_2_ to 80% confluence. The old medium was completely removed and 1 mL temperature-equilibrated osteogenic differentiation medium was added to each well for osteogenic differentiation induction. Cells cultured in DMEM were used as non-induced controls. Medium changes were performed every 3–4 days with both induced and non-induced BM-MSCs on the same schedule.

### Alkaline phosphatase (ALP) staining

BM-MSCs were maintained in an osteogenic differentiation induction medium for 14 days and osteoinductivity was evaluated by ALP staining assay using an ALP staining kit (GeFan Biotechnology, Shanghai, China). Briefly, cells were washed gently with PBS and fixed with Fixing Solution at room temperature, followed by incubating with freshly prepared ALP Staining Solution in the dark for 30 min. Cells were washed with PBS three times and observed under a light microscope (Leica DMIRB, Germany). BM-MSCs cultured in NM for 14 days were used as a negative control.

### Alizarin red S (ARS) staining and quantification

BM-MSCs were cultured in an osteogenic differentiation induction medium for 14 days and calcified nodules were detected by performing ARS staining assay. Briefly, BM-MSCs were fixed with 4% paraformaldehyde and stained with freshly prepared Alizarin Red S solution at pH 4.2 (Sigma, St Louis, MO, USA) at room temperature for 30 min. Cells were washed with PBS three times and observed under a microscope. BM-MSCs cultured in NM for 14 days were used as a negative control.

### IF staining

After treatment, BM-MSCs were seeded on glass coverslips in a 12-well plate and further cultured in an osteogenic differentiation medium for 3 days. IF staining assay was performed to detect the expression of RUNX2. Briefly, cells were fixed with 4% formaldehyde in PBS at room temperature, followed by permeabilization using 0.1% Triton X-100. Cells were rinsed three times in PBS and nonspecific binding was blocked with 1% bovine serum albumin (BSA) in PBST (PBS + 0.1% Tween 20). Diluted anti-RUNX2 primary antibody (1:1000, Abcam) was added to the cells above, followed by incubation at 4 °C overnight. Cells were washed three times with PBST and further incubated with Alexa Fluor 594-conjugated secondary antibody for 1 h at room temperature in the dark. Nuclear counterstaining was conducted by incubating the cells with 300 nM 4′,6-diamidino-2-phenylindole (DAPI) (Sigma-Aldrich, St. Louis, MO, USA) in the dark for 5 min at 37 °C. The excess staining solution was removed and slides were mounted with a drop of mounting medium after being washed three times in PBS. Fluorescence images were obtained under a Nikon confocal microscope (Eclipse TE2000U).

### RNA extraction and qRT-PCR

Total RNAs were extracted from tissue and cell samples using Trizol Reagents (Invitrogen, USA) and miR-135a-5p was extracted using mirVana^TM^ miRNA Isolation Kit (Ambion, Austin, TX) in accordance with the user’s guide. After determining RNA purity and concentration by UV spectrometry, 2 μg total RNA was reverse transcribed into complementary DNA (cDNA) using SYBR Premix Ex Taq (Takara, Dalian, China). Relative expression of circ_AFF4, FNDC5, ALP, BMP4, RUNX4, Spp1, and Colla1 was calculated by the 2^−ΔΔCt^ method and normalized to GAPDH expression. Relative expression of mature miR-135a-5p was detected by TaqMan MicroRNA Reverse Transcription Kit (Thermo Scientific, Waltham MA) using U6 small nuclear RNA (U6 snRNA) as an internal reference. The qRT-PCR data were monitored by the Applied Biosystems QuantStudio 6 Pro Real-Time PCR System (Thermo Fisher Scientific) and longitudinal analyses of these data were performed by Analysis Software 2.5 according to the manufacturer’s instructions. The qRT-PCR primers for circ_AFF4, miR-135a-5p, FNDC5, ALP, BMP4, RUNX4, Spp1, and Colla1 were synthesized by Shenggong Ltd. Co. (Shanghai, China) and their sequences were listed in Table 1.

**Table 1:** The primers in qRT-PCR.

circ_AFF4	Forward: 5′-GCATCGGTTTCTGGTGATGT-3′
	Reverse: 5′-CGGTTCATGTTGCTTAGTTG-3′
miR-135a-5p	Forward: 5′-UAUGGCUUUUUAUUCCUAUGUGA-3′
	Reverse: 5′-AACGCTTCACGAATTTGCGT-3′
FNDC5	Forward: 5′-GTGGTGAGCTGGGATGTTCT-3′
	Reverse: 5′-GCCTGCACGTGGACTATGTA-3′
GAPDH	Forward: 5′-CGACAGCAGCCGCATCTT-3′
	Reverse: 5′-CCAATACGACCAAATCCGTTG-3′
U6	Forward: 5′-CTCGCTTCGGCAGCACA-3′
	Reverse: 5′-AACGCTTCACGAATTTGCGT-3′
ALP	Forward: 5′-GAACGTGGTCACCTCCATCCT-3′
	Reverse: 5′-TCTCGTGGTCACAATGC-3′
BMP4	Forward: 5′-AGCATGTCAGGATTAGCCGA-3′
	Reverse: 5′-TGGAGATGGCACTCAGTTCA-3′
RUNX2	Forward: 5′-CGGAATGCCTCTGCTGTTAT-3′
	Reverse: 5′-TTCCCGAGGTCCATCTACTG-3′
Spp1	Forward: 5′-CGCAGACCTGACATCCAGT-3′
	Reverse: 5′-GGCTGTCCCAATCAGAAGG-3′
Colla1	Forward: 5′-CCTGGATGCCATCAAAGTCT-3′
	Reverse: 5′-AATCCATCGGTCATGCTCTC-3′

### Biotinylated RNA pull-down assay

BM-MSCs were harvested and transferred into nuclear extraction buffer. Nuclei pellet was collected by centrifuging at 700*g* for 5 min and resuspended in lysis buffer supplemented with 200 U/mL of Ribonuclease Inhibitor and protease inhibitor cocktail (Sigma, St Louis, MO, USA). Biotinylated miR-135a-5p and the mutants with the mutated putative binding sites for circ_AFF4 or FNDC5 were generated by in vitro transcription using TranscriptAid T7 High Yield Transcription Kit (Thermo Scientific, Waltham MA), followed by purification using GeneJET RNA Purification Kit (Thermo Scientific, Waltham MA). The purified miRNAs were incubated with BM-MSCs nuclei extracts above for 1 h and then with 60 µL Streptavidin magnetic beads (Invitrogen, USA) overnight at room temperature. Magnetic beads were extensively washed and the RNAs recovered with TRIzol LS (Invitrogen, USA) were analyzed by performing qRT-PCR using specific primers.

### Dual-luciferase reporter analysis

Potential binding sites between miR-135a-5p and circ_AFF4 were analyzed by using TargetScan and Starbase web tools, whereas targeted binding between FNDC5 and miR-135a-5p was analyzed by using the RNAInter web tool. circ_AFF4 wild-type (circ_AFF4-WT), circ_AFF4 mutant carrying the mutated binding sites for miR-135a-5p (circ_AFF4-MUT), FNDC5 wild-type (FNDC5-WT), FNDC5 mutant carrying the mutated binding sites for miR-135a-5p (FNDC5-MUT) were subcloned into luciferase reporter vector pMIR-report. The mutant constructs were produced using a QuickChange site-directed mutagenesis kit (Stratagene, CA, USA). BM-MSCs were seeded on a 96-well plate and grown to 70% confluence. Cells were co-transfected with Luciferase reporter vectors above, miR-135a-5p mimics, or their negative controls (miR-NC) using Lipofectamine 2000 (Invitrogen, USA). Cells were harvested and lysed after 48 h. Firefly and Renilla luciferase activities were measured using the Dual-Luciferase Reporter Assay System (Promega, Madison, WI, USA).

### RNA-immunoprecipitation (RIP)

We performed an RIP experiment using EZ-Magna RIP RNA-Binding Protein Immunoprecipitation Kit (Millipore, MA, USA) by following the manufacture’s protocol. Briefly, we transfected HEK-293T with circ_AFF4-containing adenovirus, and cells were harvested after 48 hours. Nuclei were pelleted by centrifugation at 2500*g* for 10 min and chromatin was shorn using a Dounce homogenizer. The nuclear membrane was pelleted by centrifuging at 13000 *g* for 10 min and magnetic beads conjugated with human anti-Argonaute2 (Ago2) antibody (1:50, Millipore) were added to the lysate supernatant. After incubation at 4 °C with gentle rotation for 10 h, beads were pelleted by centrifuging at 2500 rpm for 1 min, and contaminating proteins were degraded by incubating the beads with Proteinase K containing RIP buffer. Nuclear lysate incubated with human IgG antibody (Millipore) was used as a control. qRT-PCR was performed to analyze the immunoprecipitated RNA eluted from the beads.

### Fluorescence in situ hybridization (FISH)

The FISH experiment was performed to examine whether circ_AFF4 and miR-135a-5p were co-localized in the BM-MSCs cytoplasm. BM-MSCs were grown to 80% confluence and cytoskeletal structures were fixed with 4% PFA. The fixed cells were permeabilized using 0.1% Triton X-100 and incubated with circ_AFF4 or miR-135a-5p-specific probes at 37 °C, in standard hybridization buffer (900 mM NaCl, 20 mM Tris/HCl, 0.01% sodium dodecyl sulfate and 40% formamide). The fluorescently labeled circ_AFF4 or miR-135a-5p-specific probes were designed by Shanghai Invitrogen Biotechnology (Shanghai, China). Nuclei were counterstained using 1 µg/mL DAPI. The fluorescence signals were detected under a Nikon confocal microscope (Eclipse TE2000U).

### Co-immunoprecipitation (Co-IP)

Specific interaction between Irisin and Integrin αV was identified by performing the co-IP assay. Briefly, BM-MSCs were harvested by trypsinization and lysed in ice-cold immunoprecipitation buffer containing 20 mM DNase I (Sigma, St Louis, MO, USA) and protease inhibitor cocktail. After centrifuging at 15,000 × *g* for 15 min, 500 µL clarified supernatant was incubated with 5 µg antibody against Irisin or Integrin αV at 4 °C overnight, and incubated with 100 µL Protein A Agarose beads (Thermo Scientific, Waltham MA) for 2 h at room temperature. The complex-bound resin was washed with 500 µL buffer and the co-immunoprecipitated complexes were released by boiling in Lamaelli buffer (Bio-Rad, Hercules CA) at 95 °C. Co-IP products were analyzed by sodium dodecyl sulfate-polyacrylamide gel electrophoresis (SDS-PAGE) and WB.

### Protein extraction and western blot

BM-MSCs were lysed in ice-cold RIPA lysis buffer supplemented with protease inhibitor cocktail and insoluble debris was removed from the supernatant lysate by centrifuging at 15,000 × *g* for 15 min. Protein concentration was examined using BCA Protein Assay (Thermo Scientific, Waltham MA). 50 μg of total protein was loaded in each lane of 10% SDS-PAGE and transferred to nitrocellulose membranes. After the non-specific binding was blocked using 3% non-fat milk in TBST, membranes were incubated with primary antibodies against ALP (1:1000 dilutions, Abcam, UK), BMP4 (1:20000 dilutions, Abcam, UK), RUNX2 (1 µg/ml, Abcam, UK), Spp1 (1.25 µg/ml, Abcam, UK), Colla1 (1 µg/ml, Abcam, UK), FNDC5 (1:3000 dilutions, Abcam, UK), Irisin ((1:3000 dilutions, Abcam, UK), integrin αv (1:5000 dilutions, Abcam, UK) and GAPDH (1:1000 dilutions, Abcam, UK) overnight at 4 °C. Membranes were then washed three times with 0.1 M PBST, followed by incubation with horseradish peroxidase (HRP)-conjugated secondary antibodies for 1 h at room temperature. Blots were developed and detected using Pierce ECL chemiluminescent substrates (Thermo Scientific, Waltham, MA).

### In vivo bone formation assay

The animal experiments were approved by the University of South China (Hengyang, China) and performed in strict accordance with relevant guidelines. Briefly, BM-MSCs were transfected with lentiviruses carrying si-circ_AFF4, negative control or empty viruses and maintained in OM for 7 days. Bioceramic scaffolds containing hydroxyapatite and β-tricalcium phosphate (β-TCP) mixture (National Engineering Research Center for Biomaterials, Sichuan University, China) were washed with PBS, dried at 37 °C overnight, and added to the BM-MSCs for 2 h incubation. Eight-week-old female BALB/c mice were anesthetized with 5% isoflurane and BM-MSCs/bioceramic composites were subcutaneously injected into the dorsal side of mice (four implants per animal). Mice without implantation were used as the negative control. Eight weeks after the implantation mice were sacrificed and samples were harvested, followed by fixation with 4% paraformaldehyde (PFA) in PBS for 2 h.

### Hematoxylin and eosin (H&E) staining

Dissected mouse femurs were decalcified with 14% ethylenediamine tetra-acetic acid (EDTA) and embedded in paraffin. For H&E staining, bone samples were sliced into coronal sections of 5 μm in thickness and deparaffinized using three changes of xylene. After stepwise rehydration with decreasing concentration of ethanol, samples were incubated with hematoxylin for 5 min and the excess stain was removed by rinsing with 1% acid ethanol. Slides were washed under running tap water and incubated with 0.5% eosin for 1 min, followed by stepwise dehydration with increasing concentration of ethanol and three changes of xylene. A drop of Permount (VWR, South Plainfield, NJ, USA) was added to the slide, and images were captured under a microscope (Leica DMIRB, Germany).

### Masson staining

Masson staining was performed using Trichrome Stain Kit (Sigma, St Louis, MO, USA) by following the manufacturer’s protocol. Briefly, bone sample slides were deparaffinized using three changes of xylene and incubated with Bouin’s Solution overnight at room temperature. After the excessive yellow color was washed away under running tap water, slides were stained with Working Weigert’s Iron Hematoxylin Solution for 5 min, followed by incubation in Biebrich Scarlet-Acid Fuchin for 5 min and in Working Phosphotungstic/Phosphomolybdic Acid Solution for. Next, slides were placed in Aniline Blue solution for 8 min and in 1% acetic acid for 2 min, before stepwise dehydration with increasing concentration of ethanol and three changes of xylene. A drop of Permount was added to the slide and images were captured under a microscope.

### Immunohistochemistry (IHC)

After fixation, bone samples were embedded in paraffin, cut into sections at 5 µm using a cryostat, and mounted onto histological slides which were coated with gelatin. Slides were dried overnight, deparaffinized using three changes of xylene, and rehydrated with decreasing concentration of ethanol. Nonspecific binding was blocked with 1% BSA and a diluted primary antibody against RUNX2 (1:1000 dilutions, Abcam, UK) was applied to the slides at 4 °C overnight. Slides were washed three times with TBST and incubated with HRP-conjugated secondary antibody at room temperature in dark for 1 h. For nuclear counterstain 300 µl of diluted DAPI was added to each slide and incubated for 3 min at room temperature. Images were obtained under a fluorescence microscope.

### Statistical analysis

Statistical analyses were performed by using SPSS software (version 17.0; Chicago, IL, USA). All experiments were repeated at least three times independently and data were presented as the mean ± standard deviation. Differences between two experimental groups were analyzed by Student’s *t* test, and differences among more than two groups were evaluated by one-way analysis of variance (ANOVA). *P* value < 0.05 was considered statistically significant.

## Supplementary information

Supplementary figure legends

Figure S1

Figure S2

Figure S3

Figure S4

author contribution form

## Data Availability

All data generated or analyzed during this study are included in this article. The datasets used and/or analyzed during the current study are available from the corresponding author on reasonable request.
