# Supplementary material for: Circular RNA AFF4 modulates osteogenic differentiation in BM-MSCs by activating SMAD1/5 pathway through miR-135a-5p/FNDC5/Irisin axis
Source: Cell Death Dis. 2021 Jun 18;12(7):631. doi: 10.1038/s41419-021-03877-4 (PMC8213698; doi:10.1038/s41419-021-03877-4)
Supplement: Supplementary file 1 — Supplementary figure legends [file 41419_2021_3877_MOESM1_ESM.docx]

**Supplementary figure legends**

**Figure S1**

**
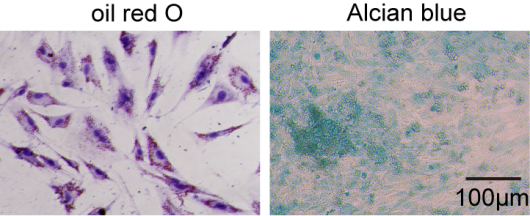
**

**Figure S1.** Representative images of adipocyte and chondrocyte differentiation of BM-MSCs analysed using cytochemical staining with oil red O and Alcian blue.

**Figure S2**


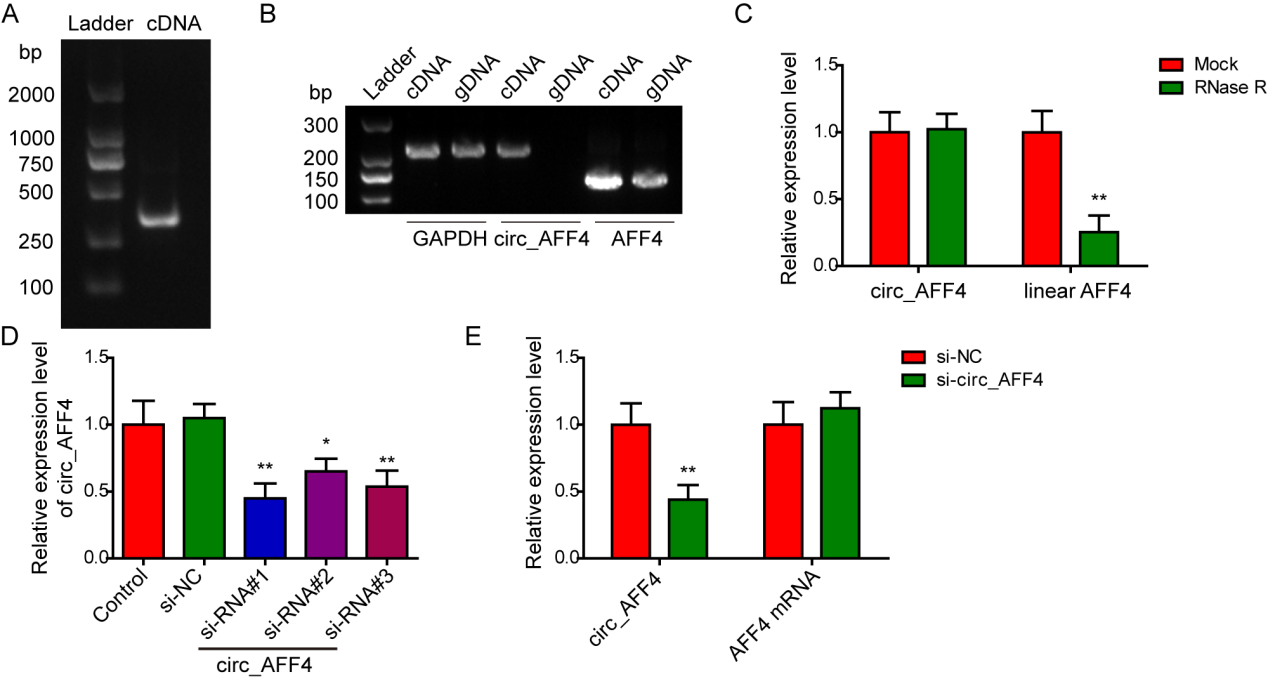


**Figure S2.** (A) **q**RT-PCR product of circ_AFF4 was validated by agarose gel electrophoresis. (B) qRT-PCR assay showed the expression of circ_AFF4 and AFF4 mRNA in BM-MSCs administered with RNase R or Mock control. (C) qRT-PCR showed the expression levels of circ_AFF4 and linear_AFF4 in BM-MSCs after the transfection of si-NC or si-circ_AFF4. (D) The expression of circ_AFF4 was detected using qRT-PCR assay. (E) qRT-PCR assay was subjected to determine the expression levels of circ_AFF4 and AFF4 mRNA. **P*<0.05, ***P*<0.01.

**Figure S3**


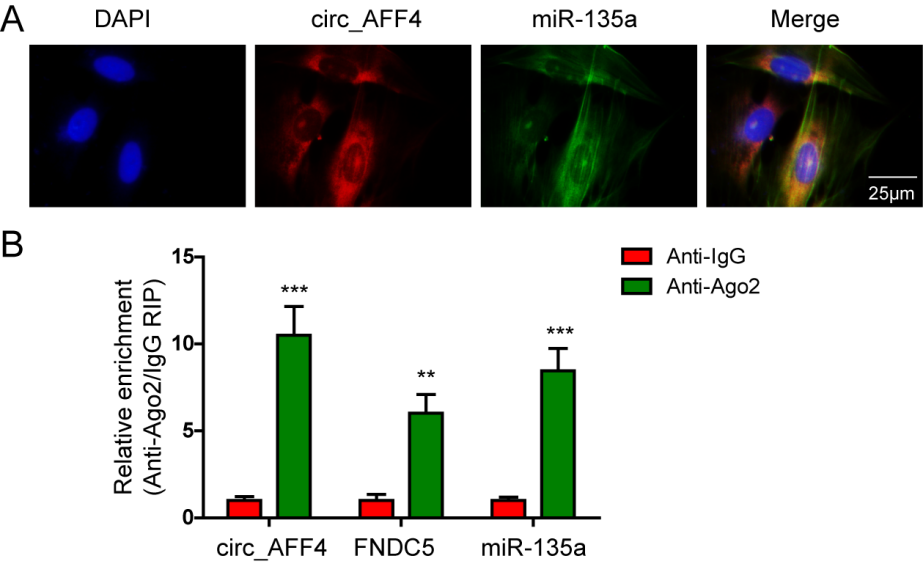


**Figure S3.** (A) RNA-FISH was used to determine the co-localization of circ_AFF4 and miR-135a. (B) RIP assay demonstrated that circ_AFF4 was enriched in Ago-containing immune-precipitates. ***P*<0.01 and ****P*<0.001.

**Figure S4**


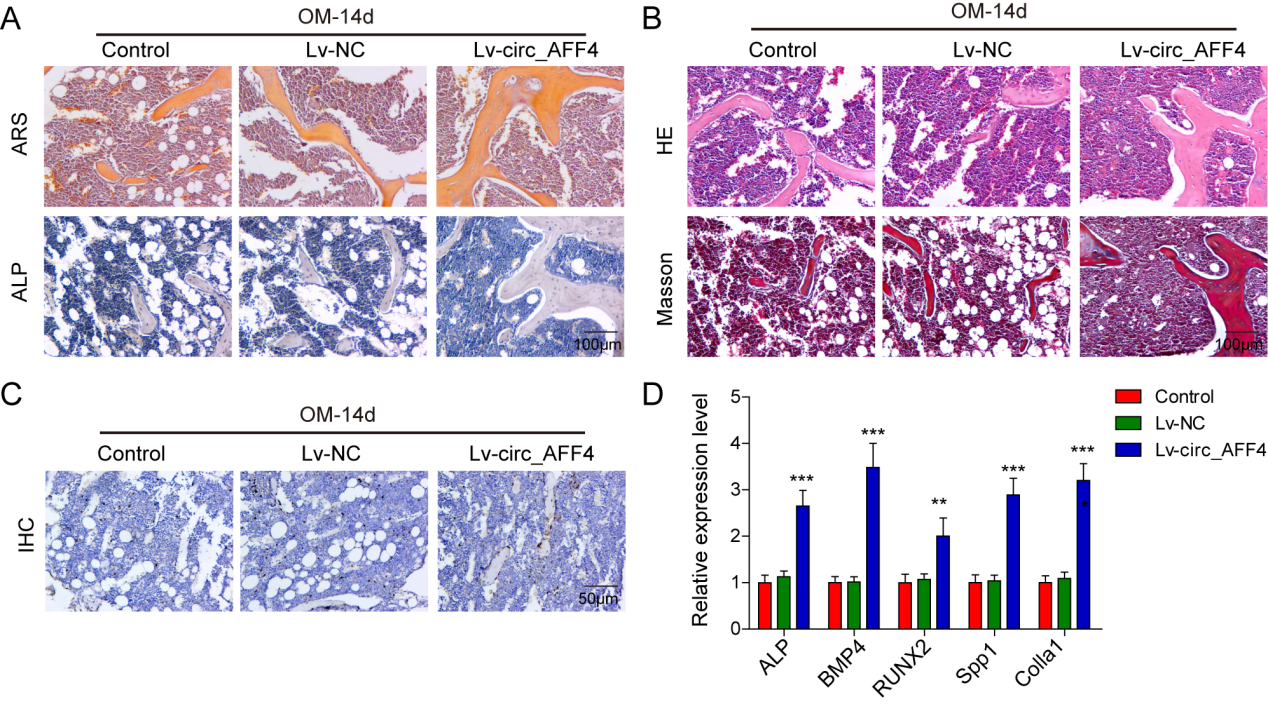


**Figure S4.** (A) ARS and ALP staining assays were performed to compare the bone cell formation in mice treated with Lv-circ_AFF4 or Lv-NC. (B) Representative images of H&E and Masson staining in mouse femurs showing the *in vivo* bone formation and collagen deposition respectively. (C) Expression of the osteogenic differentiation-related transfection factor RUNX2 was evaluated using IHC staining. (D) qRT-PCR was conducted to determine the expression levels of osteogenic marker genes ALP, BMP4, RUNX2, Spp1 and Colla2 in femur samples from indicated mice. The data were shown as mean ± SD based on at least three independent experiments. ***P*<0.01 and ****P*<0.001.
