# Supplementary figures and images for: Circular RNA AFF4 modulates osteogenic differentiation in BM-MSCs by activating SMAD1/5 pathway through miR-135a-5p/FNDC5/Irisin axis
Source: Cell Death Dis. 2021 Jun 18;12(7):631. doi: 10.1038/s41419-021-03877-4 (PMC8213698; doi:10.1038/s41419-021-03877-4)

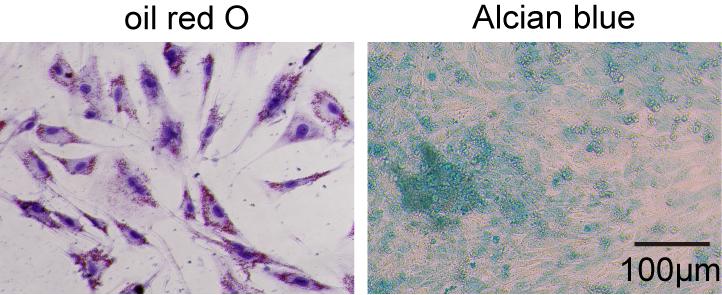

Supplement: Supplementary file 2 — Figure S1 [file 41419_2021_3877_MOESM2_ESM.jpg]

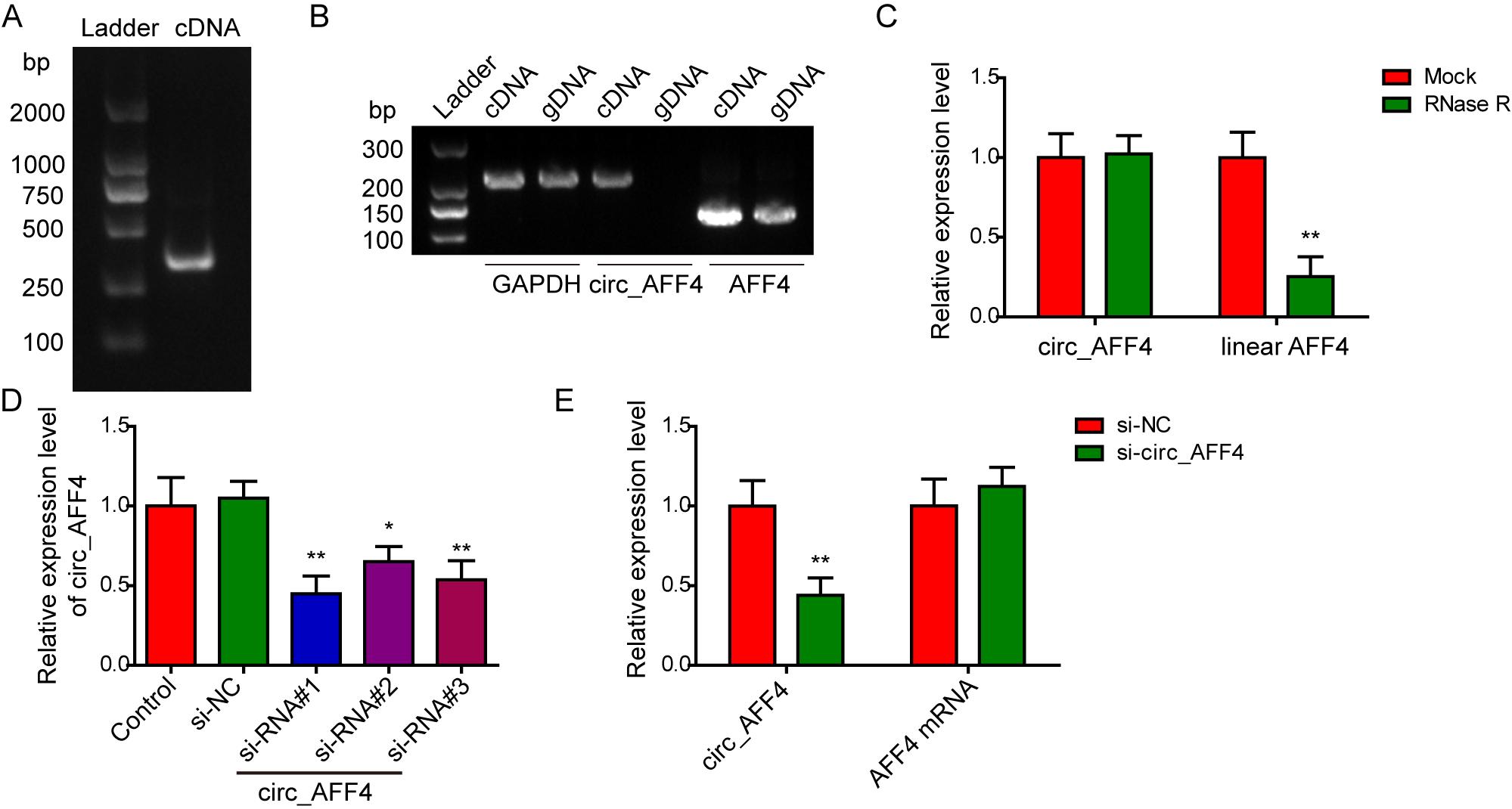

Supplement: Supplementary file 3 — Figure S2 [file 41419_2021_3877_MOESM3_ESM.jpg]

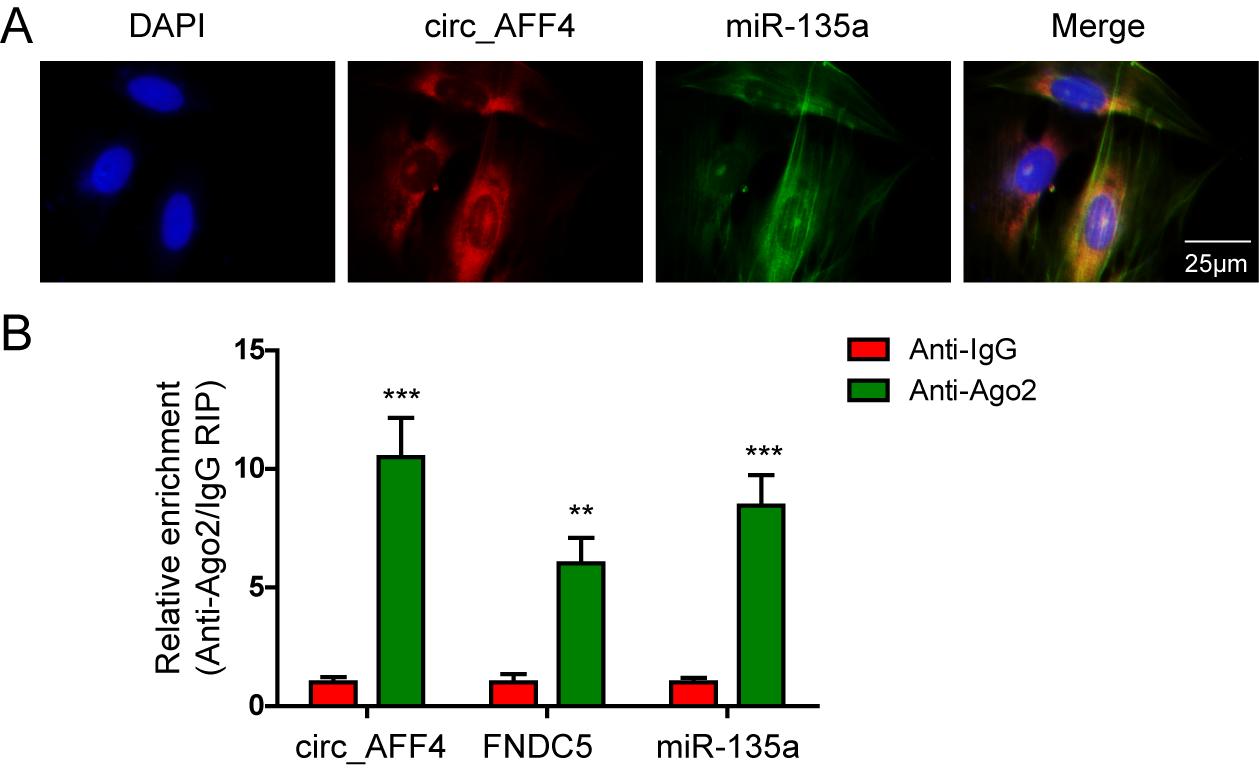

Supplement: Supplementary file 4 — Figure S3 [file 41419_2021_3877_MOESM4_ESM.jpg]

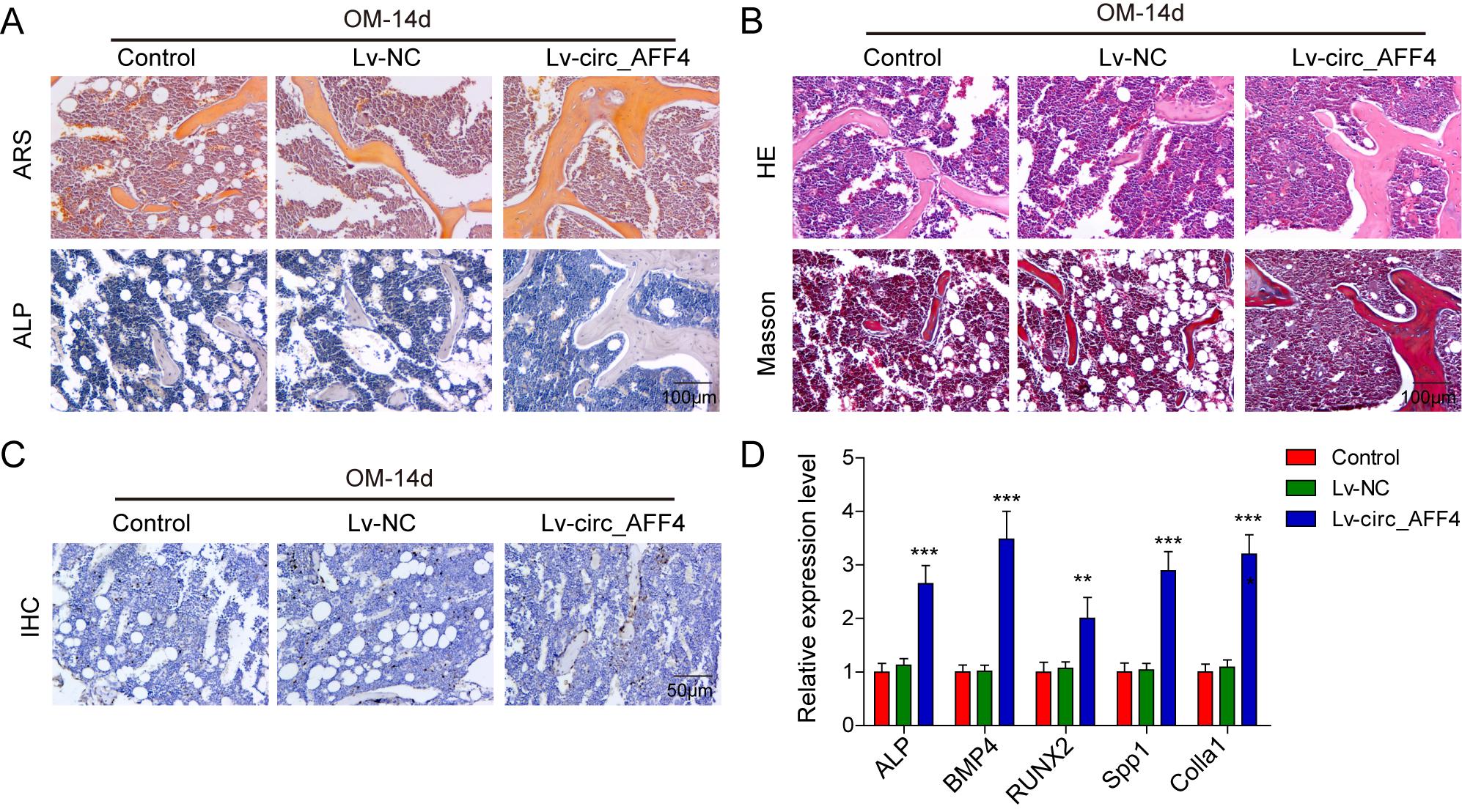

Supplement: Supplementary file 5 — Figure S4 [file 41419_2021_3877_MOESM5_ESM.jpg]

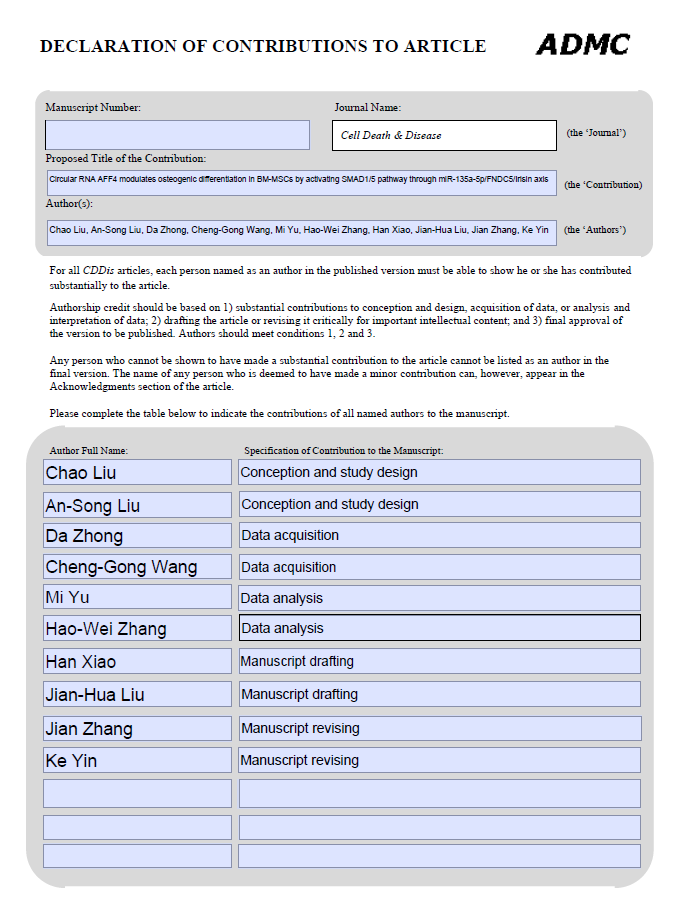

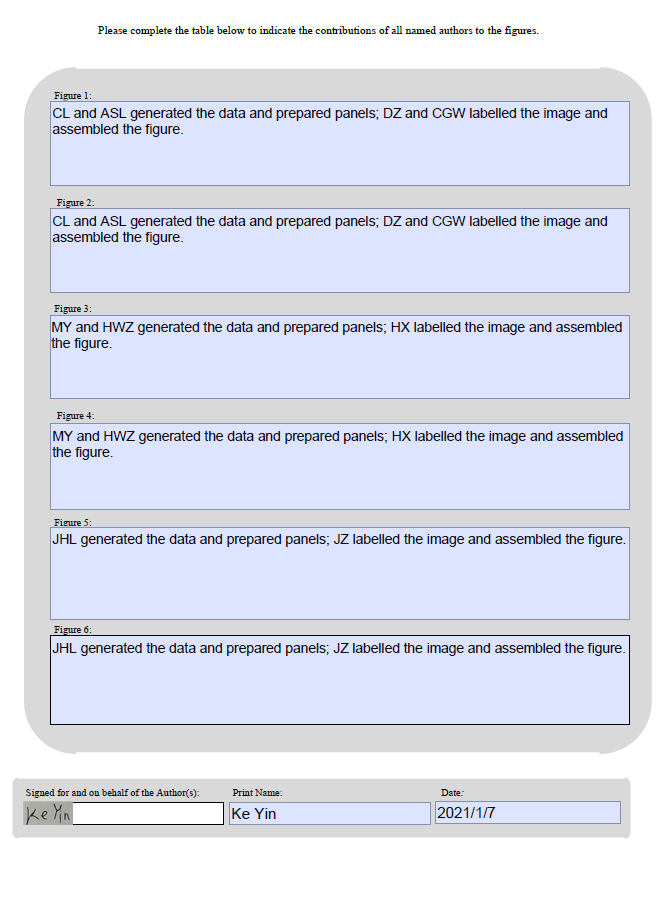

Supplement: Supplementary file 6 — author contribution form [file 41419_2021_3877_MOESM6_ESM.doc]
